# Identification of Pancreatic Ductal Adenocarcinoma Extracellular Matrix Signatures from In-Depth Proteomic Profiling that Correlate with Lymphocyte Infiltration

**DOI:** 10.1158/2767-9764.CRC-25-0460

**Published:** 2026-06-05

**Authors:** James M. Considine, Dharma Pally, Sarah A. O’Brien, Jessica N. Egan, Di Feng, Jeanine Pignatelli, Abhishek Kashyap, Nikita S. Sharma, Alexandra Naba

**Affiliations:** 1 Department of Physiology and Biophysics, University of Illinois Chicago, Chicago, Illinois.; 2 Oncology Discover Research Department, Boehringer Ingelheim, Ridgefield, Connecticut.; 3 Global Computational Biology and Data Science Department, Boehringer Ingelheim, Ridgefield, Connecticut.; 4 University of Illinois Cancer Center, Chicago, Illinois.

## Abstract

The extracellular matrix (ECM) is a complex assembly of proteins surrounding cells. It is a critical component of the tumor microenvironment (TME) that plays an active role in tumor progression and modulation of tumor response to treatment. Pancreatic ductal adenocarcinoma (PDAC) is a cancer type characterized by one of the worst prognoses, as it is often diagnosed at an advanced stage. It is also characterized by a very dense ECM, which hinders efficient drug delivery. In addition, PDACs are considered “cold” tumors as they fail to elicit a strong immune response, challenging the use of immunotherapy for patients with PDAC cancer. Yet, the interplay between the ECM and immune cells within the PDAC TME remains poorly understood. In this study, we used ECM-focused proteomics to profile the ECM compositions of PDAC mouse models characterized by different levels of CD8^+^ T-cell infiltration. We found that CD8^lo^, or “cold” tumors, and CD8^hi^, or “hot” tumors, exhibited different ECM profiles. Interrogation of publicly available single-cell RNA sequencing datasets of human PDACs further revealed that the ECM proteins distinguishing hot and cold PDACs are secreted by multiple stromal cell populations, including cancer-associated fibroblasts, stellate cells, and macrophages. Last, we found that the expression of a subset of the genes encoding ECM proteins characteristic of the CD8^lo^ phenotype correlated with CD8^+^ T-cell infiltration in human PDAC samples and patient survival. This study paves the way for the development of ECM-modulating interventions to enhance immune cell infiltration and responsiveness to immunotherapy.

**Significance:**

We report the identification of ECM protein signatures correlating with the level of CD8^+^ lymphocyte infiltration in murine models of PDACs and human samples. This work paves the way for the development of ECM-modulating therapeutic strategies to enhance lymphocyte infiltration and, hence, the efficacy of immunotherapies.

## Introduction

Over the past two decades, significant advances in immunotherapy, a therapeutic modality that aims to stimulate a patient’s immune system—and in particular cytotoxic CD8^+^ lymphocytes (or CD8^+^ T cells)—to attack its tumor, have transformed the lives of countless patients with cancer (1–5). These advances have been facilitated by an increased appreciation for the importance of the tumor microenvironment (TME), that is, the non–tumor cell components of a tumor, in cancer progression and response to therapy (6–8).

A prominent component of the TME is the extracellular matrix (ECM). The ECM is a complex and dynamic assembly of more than 150 proteins that constitutes a scaffold providing physical and mechanical support and signaling cues to cells (9). Most tumors are characterized by increased ECM content, or desmoplasia, and we know that changes in ECM composition or architecture affect all steps of cancer progression, from supporting tumor cell proliferation and survival to promoting tumor angiogenesis and enhancing cell invasion and dissemination (10–12). We also now have evidence that the tumor ECM composition can be altered by anticancer treatments (13). Conversely, the ECM can influence the composition of the TME, in particular the immune microenvironment (14, 15), and hence can modulate response to anticancer treatment (14, 16, 17). Thus, intervening on the ECM could increase the efficacy of anticancer therapies, including immunotherapy (18).

Pancreatic ductal adenocarcinoma (PDAC) is a cancer type characterized by one of the worst prognoses, as it is often diagnosed at an advanced stage. The American Cancer Society projects that this cancer will claim the lives of more than 50,000 patients in the United States in 2025 (19). PDAC is notoriously characterized by excessive ECM accumulation and high stromal cell content [including cancer-associated fibroblasts (CAF), stellate cells, and inflammatory cells]. A recent study has shown that ECM content negatively correlates with PDAC patient survival [median survival of patients with ECM^hi^ tumors: 15.3 months vs. 22.9 months for patients with ECM^lo^ tumors (*P* = 0.02; ref. 20). A series of studies by Tian and colleagues (21–23) using patient samples and mouse models of PDACs has identified subsets of ECM proteins correlating with PDAC progression and metastatic dissemination. It has thus been proposed that targeting components of the PDAC stroma by directly modulating the ECM or the cells contributing to the production of the ECM could offer long-awaited therapeutic benefits (24–27). Importantly, PDACs are often considered “cold,” i.e., as failing to elicit a strong immune response, challenging the use of immunotherapy. Studies have also shown that a higher level of T-cell infiltration has been correlated with better patient outcome (28–32). One of the factors often invoked to explain this phenotype is the density of the ECM of PDAC that could act as a barrier to immune cell infiltration, although this hypothesis has now been challenged (31). Because of the demonstrated interplay between the tumor stroma, the ECM, and lymphocytes, and the successes of immunotherapies, it thus becomes appealing to attempt to modulate the PDAC stroma and the ECM to convert a cold TME into a hot one and render these tumors responsive to immunotherapy (33–35). However, to do so, we first must identify relevant ECM targets.

In this study, we used proteomics to profile the ECM composition of *KRAS*-driven and translationally relevant murine models of CD8^hi^ (“hot”) and CD8^lo^ (“cold”) PDACs. Using highly stringent identification criteria, we report the identification of a 16-ECM-protein signature characteristic of a cold phenotype and an 8–ECM protein signature correlating with a hot tumor phenotype. We validated these findings using immunohistochemistry (IHC), which provides additional insights into the distribution pattern of ECM proteins within the PDAC TME. Leveraging publicly available single-cell RNA sequencing (scRNA-seq) datasets generated on human PDAC samples, we further demonstrate that the genes encoding signature ECM proteins are expressed by multiple cell populations of the PDAC TME and that their expression levels correlated with CD8^+^ T-cell infiltration. Last, we show that the expression of a subset of the genes encoding ECM proteins characteristic of the CD8^lo^ phenotype correlated with patient survival. Our work paves the way for the development of matritherapies (36) that will aim to modulate the composition of the ECM toward promoting the recruitment and/or the activation of antitumor immune cells and, hence, increase immunotherapy efficacy. Importantly, with an in-depth characterization of the matrisome of hot PDACs, we can also begin to design approaches using ECM proteins for the targeted delivery and anchoring of therapeutic payloads for enhanced efficacy and reduced side effects.

## Materials and Methods

### Cell lines and animal studies

All animal studies were approved by Boehringer Ingelheim’s Institute for Animal Care and Use Committee (protocol number 19-636-E). Female C57BL/6 mice were purchased from The Jackson Laboratory and used at 6 to 8 weeks of age. KPCY TC3 (6419c5), KPCY TC4 (6422c1), KPCY TH2 (2838c3), and KPCY TH3 (6499c4) cell lines are derived from a previously established autochthonous mouse model of PDAC (*Kras*^*LSL-G12D/+*^; *Trp53*^*LSL-R172H/+*^; *Pdx1-Cre*; *Rosa26*^*YFP/YFP*^, referred to as “KPCY PDAC”; refs. 37, 38) and were acquired from Dr. Stanger at the University of Pennsylvania in September 2020. The KPCY cell lines were cultured in DMEM (Corning #10-017-CV) supplemented with 10% heat-inactivated FBS (Genesee Scientific #25-514H), 1% GlutaMAX (Gibco #35050061), and 1X penicillin/streptomycin (Gibco #15140-122). Cells were plated in T75 flasks and passaged for three passages prior to implant into mice at passage 13 to 15. Cell lines were routinely tested for *Mycoplasma* and other pathogens through the IDEXX IMPACT 3 panel (#41-00041). The cell lines obtained directly from the laboratory that generated them were not reauthenticated. For mouse tumor studies, 3 × 10^5^ cells were injected subcutaneously in the right flank. Tumors were measured three times per week, and tumor volume was calculated using the following formula: [(length × width^2^ × π)/6]. Tumors were harvested at 500 mm^3^ to compare immune infiltration by immunostaining and harvested at >100 mm^3^ and snap-frozen in liquid nitrogen for subsequent proteomic analyses.

### Histological staining and analysis

Mouse tumors were fixed in formalin and embedded in paraffin according to standard procedures. Sections (5 μm) were mounted on positively charged slides, dried, and baked at 60°C for an hour. Sections were deparaffinized using xylene and rehydrated by incubation in solutions of descending ethanol concentrations and then in tap water. Consecutive sections were stained with the following stains.

#### Masson’s trichrome staining

Deparaffinized sections were incubated in Bouin’s fixative (StatLab, #FXBOULT) for 1 hour at 56°C, rinsed with tap water, stained with Biebrich Scarlet solution (Electron Microscopy Sciences, #26033-25) for 8 minutes at room temperature, washed in distilled water, incubated in phosphotungstic/phosphomolybdic acid (Electron Microscopy Sciences, #2636705) for 15 minutes, and finally, stained with Aniline Blue solution (Electron Microscopy Sciences, #2636706) for 10 minutes. For each tumor (*n* = 5 per tumor group), two independent sections at least 50 μm apart were stained to account for possible heterogeneity in collagen content within tumors.

#### Picrosirius red staining

Deparaffinized sections were incubated in Bouin’s fixative (Mercedes Scientific, #EKI 22771L) for 1 hour at 60°C, rinsed with tap water, stained with 0.1% Fast Green counterstain for 15 minutes at room temperature, incubated with 1% acetic acid for 4 minutes, and finally, stained with Picrosirius red solution (Mercedes Scientific, #SRS999) for 30 minutes. Slides were briefly rinsed in distilled water, dehydrated, and mounted with Micromount (Leica Microsystems, #3801730). For each tumor (*n* = 5 per tumor group), two independent sections at least 50 μm apart were stained to account for possible heterogeneity in collagen content within tumors.

#### Immunohistochemistry (IHC)

The following antibodies were used: anti–α smooth muscle actin (αSMA), anti-CD31, anti-CD4, anti-CD8, anti-F4/80, anti-Ly6G, anti–collagen VIII (COL8A1), anti–fibulin 5, anti-MMP9, and anti-TGFβ induced. Catalog numbers, Research Resource Identifiers, concentrations, and epitope retrieval conditions used are provided in Supplementary Table S1. The number of sections and of tumors stained is indicated in the figure legends. Staining was performed using the BOND Polymer Refine Detection Kit (Leica, #DS9800) on the BOND RX automated stainer (Leica Biosystems) according to a standard preset protocol. After deparaffinization, sections were subjected to heat-induced epitope retrieval. Endogenous peroxidase activity and nonspecific binding sites were blocked by sequentially treating samples with peroxidase block (BOND Polymer Refine Detection Kit) and protein block (Background Sniper, Biocare Medical, #BS966) for 15 minutes at room temperature. Sections were then incubated with the primary antibody for 30 minutes. After several washes, signal detection was performed with a horseradish peroxidase–coupled anti-rabbit secondary antibody and 3, 3′-diaminobenzidine (DAB; BOND Polymer Refine Kit) by incubating the sections for 15 and 10 minutes at room temperature, respectively. Tumor sections were counterstained with hematoxylin. All slides were dehydrated in an Autostainer XL, mounted with Micromount (Leica Biosystems), and scanned at a resolution of 40× on a Leica Aperio AT2 whole-slide scanner for qualitative assessment. Representative full-section scans for each staining are available via the repository Figshare at https://doi.org/10.6084/m9.figshare.30687044.

#### Histologic analysis using HALO

All slides were scanned at a resolution of 40× on a Leica Aperio AT2 whole-slide scanner. Each tumor section was manually annotated using Aperio ImageScope (v.12.4.6.5003) to exclude artifacts such as tissue folds or necrotic areas, and fully annotated tumor sections were imported to HALO (Indica Labs) and analyzed as described below.

##### Determination of immune cell density

Hematoxylin and DAB stains were manually assigned in the algorithm, and thresholds were adjusted to detect and differentiate nuclei and different populations of immune cells: CD8^+^ T-cells, CD4^+^ T cells, F4/80^+^ macrophages, or Ly6G^+^ neutrophils. After obtaining the number of immune cells for each tumor section, we calculated cell density by dividing the number of immune cells by the tumor area in mm^2^. Representative images of the HALO analysis for CD8^+^ T cells are provided in Supplementary Fig. S1. Statistical analysis was performed using the Welch *t* test, assuming unequal variance for all conditions.

##### Quantification of αSMA^+^ and CD31^+^ staining

The αSMA^+^ or CD31^+^ staining area was determined using the HALO area quantification algorithm (v2.3.1), which involved first defining the settings for the hematoxylin counterstain and then setting thresholds to detect the αSMA- or CD31-positive areas, categorized as presenting weak, moderate, and strong signals. The results were expressed as total area of tissue analyzed, area of positive staining, and percentage of positive area, which are normalized measures considering the total tissue area analyzed for each slide. Of note, mural cells lining the blood vessel wall can also be αSMA^+^, and ideally one would subtract the CD31 mask from that of the αSMA to achieve the most accurate quantification of CAFs or use additional CAF markers.

##### Determination of tumor collagen content

The HALO area quantification algorithm was used to quantify the percentage of collagen area in tumor sections stained with either Masson’s trichrome or Picrosirius red. Color channels were manually assigned by selecting single-stained regions. A third channel was added to recognize and remove artifactual stains (black or brown) and was labeled “exclusion stain.” Thresholds were set manually for the two primary colors in each image. Two phenotypes were defined: a tissue-positive region where collagen staining is negative but counterstain is positive, and a collagen-positive region where collagen staining is positive (blue when using Masson’s trichrome and green when using Picrosirius red). Collagen content is calculated by dividing the collagen-positive area by the total tumor area (i.e., tissue-positive and collagen-positive regions). Statistical analysis was performed using the Welch *t* test, assuming unequal variance for all conditions.

### Sample preparation for proteomic analysis

#### Tissue decellularization

Tissues (40 to 120 mg) were homogenized using an OMNI Bead Ruptor using the 8 m/second setting with three 10-second cycles and a 60-second break between each cycle. The bead mix used for tissue homogenization contained four 2.8-mm ceramic beads (OMNI International #19628) and four 2.4-mm metal beads (OMNI International #19-610). ECM enrichment was achieved through the sequential extraction of intracellular proteins in order of decreasing protein solubility, using a subcellular protein fractionation kit for tissues following the manufacturer’s instructions (Thermo Fisher Scientific, #87790) as previously described (39). The efficiency of the sequential extraction of intracellular components and concomitant ECM protein enrichment was monitored by Western blot analysis using the following antibodies: anti–collagen I (Sigma, #AB765P, used at a 0.5 μg/mL), anti-histone H4 (Sigma, #05-858, antibody used at 1/30,000 dilution), anti-GAPDH (Sigma, #MAB374, used at 2 μg/mL), and anti–integrin β1 serum kindly gifted by Dr. Richard O. Hynes (used at 1/1,000 dilution).

We analyzed five independent tumors for each tumor model for a total of 10 tumors with low CD8^+^ T-cell infiltration (CD8^lo^), also referred to as “cold” tumors” (TC3, *n* = 5 and TC4, *n* = 5), and 10 tumors with high CD8^+^ T-cell infiltration (CD8^hi^), also referred to as “hot” tumors (TH2, *n* = 5 and TH3, *n* = 5).

#### Protein digestion for mass spectrometry analysis

ECM-enriched protein samples were subsequently solubilized and digested into peptides following an established protocol (40–42). In brief, proteins were solubilized in an 8 mol/L urea solution prepared in 100 mmol/L NH_4_HCO_3_, and protein disulfide bonds were reduced using 10 mmol/L dithiothreitol (Thermo Fisher Scientific, #A39255). Reduced disulfide bonds were then alkylated with 25 mmol/L iodoacetamide (Thermo Fisher Scientific, #A39271) for 30 minutes in the dark at room temperature. The urea concentration was brought to 2 mol/L, and proteins were then deglycosylated with PNGaseF (New England Biolabs, #P0704L) for 2 hours at 37°C and digested sequentially, first with Lys-C (Thermo Fisher Scientific, #90307) for 2 hours at 37°C and then with trypsin (Thermo Fisher Scientific, #90058), overnight at 37°C. A fresh aliquot of trypsin was added the following day and samples were incubated for an additional 2 hours at 37°C. All incubations were performed under mild agitation. Samples were acidified with 50% trifluoroacetic acid and desalted using Pierce Peptide Desalting Spin Columns, as previously described (43). Peptides were lyophilized and then reconstituted in a solution containing 95% HPLC-grade water, 5% acetonitrile (ACN), and 0.1% formic acid (FA), and the concentration of the peptide solution was measured using the Pierce Quantitative Colorimetric Peptide Assay Kit (#23275).

#### Peptide fractionation

Forty micrograms of each sample were fractionated by high-pH reversed-phase chromatography using Shimadzu UFLC and Waters XBridge column (C18 4.6 × 150 mm, 3.5 μm). Buffer A consisted of 10 mmol/L ammonium formate, and buffer B consisted of 10 mmol/L ammonium formate with 90% ACN; both buffers were adjusted to pH 10 with ammonium hydroxide. The gradient starts with 1% B at 1 mL/minute for 3 minutes, then 1% B to 25% B in 60 minutes, 65% B in 10 minutes, and ramped to 85% B in 5 minutes. The gradient was held at 85% B for 10 minutes before being ramped back to 1% B. Eighty fractions were collected at a 1 mL/minute flow rate and combined into eight pools as follows: first, the last eight fractions (fractions 73–80) were pooled with fractions 65 to 72, with fraction 80 being combined with 72, 79 with 71, 78 with 70, etc. The volume of these pooled fractions was brought down using a SpeedVac to 1 mL each and pooled with the next set of fractions, 72 being pooled with 64, 71 with fraction 63, 70 with 62, etc. Sample volume was reduced to 1 mL, and samples were then pooled with the next set of eight fractions, 64 being pooled with fraction 56, 63 with 55, 62 with 54, etc. This process was repeated until the concatenation of 80 fractions into eight pools, each of 10 fractions, was achieved. Concatenation was followed by desalting using Nestgroup C18 tips. Fractionated peptides were dried and redissolved in 25 μL 0.1% FA, and each pool was analyzed using liquid chromatography coupled to tandem mass spectrometry (LC-MS/MS). All reagents were LC/MS-grade and purchased from Sigma-Aldrich. All solvents were Optima LC/MS-grade and purchased from Fisher Chemical.

#### LC-MS/MS data acquisition

Approximately 5 μg of pooled fractionated peptides were analyzed using a Q Exactive HF mass spectrometer coupled with an UltiMate 3000 RSLC nanosystem with a Nanospray Flex Ion Source (Thermo Fisher Scientific). The samples were loaded into a Waters nanoEase M/Z C18 (100Å, 5 μm, 180 μm × 20 mm) trap column and then a 75 μm × 150 mm Waters BEH C18 (130Å, 1.7 μm, 75 μm × 15 cm) column and separated at a flow rate of 300 nL/minute. Solvent A was 0.1% FA in water, and solvent B was 0.1% FA and 80% ACN in water. The solvent gradient of LC was 5% B in 0 to 3 minutes, 10% B at 3.2 minutes, 40% B at 55 minutes, 95% B at 60 minutes, wash 95% for 5 minutes, followed by 5% B equilibration until 75 minutes. Full MS scans were acquired in the Q-Exactive HF mass spectrometer over the 350 to 1,400 m/z range with a resolution of 60,000. The AGC target value was 1.00E + 06 for the full scan. The 15 most intense peaks with charge states 2, 3, 4, and 5 were fragmented in the HCD collision cell with a normalized collision energy of 30%; these peaks were then excluded for 30 seconds within a mass window of 1.2 m/z. A tandem mass spectrum was acquired in the mass analyzer with a resolution of 15,000. The AGC target value was 5.00E + 04. The ion selection threshold was 1.00E + 04 counts, and the maximum allowed ion injection time was 30 milliseconds for full scans and 50 milliseconds for fragment ion scans.

#### Database searching

Spectra were searched against the UniProt mouse database (0221207_UniProt mouse containing 17,138 entries) using MaxQuant with the following parameters: parent mass tolerance of 20 ppm, fragment ion mass tolerance of 20 ppm, and assuming the digestion enzyme stricttrypsin and allowing a maximum of two missed cleavage sites. Carbamidomethyl (C) of cysteine was specified as a fixed modification. Gln- > pyro-Glu of the N-terminus, deamidation of asparagine and glutamine, and oxidation of methionine, proline, and lysine were specified as variable modifications, the latter two being characteristic posttranslational modifications of ECM proteins, in particular collagens and collagen domain-containing proteins, as we previously reported (44). MaxQuant LFQ was used for label-free quantification using the default setting.

#### Criteria for protein identification and data analysis

Scaffold (version Scaffold_5.2.1, Proteome Software Inc.) was used to validate MS/MS-based peptide and protein identifications. We used stringent criteria to accept peptide and protein identification: peptide identifications were accepted if they could be established to achieve an FDR of less than 1.0% by the Percolator posterior error probability calculation (45); protein identifications were accepted if they could be established with an FDR of less than 1.0% and at least two identified peptides. Protein probabilities were assigned by the Protein Prophet algorithm (46). Proteins that contained similar peptides and could not be differentiated based on MS/MS analysis alone were grouped to satisfy the principles of parsimony. Proteins sharing significant peptide evidence were grouped into clusters.

MS output was further annotated to identify ECM and non-ECM components (40, 47). Specifically, matrisome components were classified as core matrisome or matrisome-associated components and further categorized into groups based on structural or functional features: ECM glycoproteins, collagens, or proteoglycans for core matrisome components and ECM-affiliated proteins, ECM regulators, or secreted factors for matrisome-associated components. Total precursor ion intensities were used to estimate protein abundance and conduct label-free intergroup quantification. An unpaired Student *t* test was applied to determine the statistical significance of changes in protein abundance between CD8^hi^ and CD8^lo^.

### Pathway enrichment analyses

Protein interaction analysis was performed using Search Tool for the Retrieval of Interacting Genes/Proteins (STRING v12; https://string-db.org/; ref. 48). Pathway analysis was performed using the Reactome database (https://reactome.org/; ref. 49). Transcription factor (TF) enrichment analysis was performed using ChIP-X Enrichment Analysis 3 (ChEA3; https://maayanlab.cloud/chea3/; ref. 50).

### scRNA-seq

A published scRNA-seq dataset of human PDAC (51) was retrieved from the China National Center for Bioinformation (https://ngdc.cncb.ac.cn/gsa/) with the accession number GSA: CRA001160. Scanpy 1.9 was used to analyze and visualize the scRNA-seq datasets in this study, keeping the t-SNE coordinate provided in the original publication.

### Single-sample gene set enrichment analysis and tumor immune estimation resource analyses

To examine the relationship between gene expression and immune cell infiltration, we used single-sample gene set enrichment analysis (ssGSEA; ref. 52) and the tumor immune estimation resource (TIMER) algorithm via the TIMER2.0 web server (http://timer.cistrome.org/; ref. 53). The gene module in TIMER2.0 was used to calculate the association score of each gene with CD8^+^ T cells. Purity adjustment was used to account for the influence of tumor purity on the relationship between ECM gene expression and immune cell infiltration levels in The Cancer Genome Atlas (TCGA) PAAD (PDAC) RNA-seq dataset (54). Associations were considered significant if the *P* value was less than 0.05.

### Survival analysis

Level-3 RNA-seq expression data and corresponding clinical annotations for TCGA cohorts were obtained from the Genomics Data Commons (https://portal.gdc.cancer.gov/). For each gene of interest, patients (*n* = 90) were stratified into high- and low-expression groups using the upper (75th percentile or upper quartile) and lower (25th percentile or lower quartile) quartiles to maximize biological contrast and reduce noise from intermediate expression cases (Q2–Q3). Kaplan–Meier survival curves were generated for these expression-defined subgroups, and differences in overall survival (OS) were assessed using the log-rank test. We conducted survival analyses across multiple genes of interest. For each gene, expression values were stratified into quartiles, and a multivariable Cox proportional hazards model was fitted using the lower quartile as the reference group. To account for potential confounding arising from variable tumor purity and stroma composition, the Estimation of STromal and Immune cells in MAlignant Tumors using Expression–derived stromal score was incorporated as an adjustment covariate (55). This approach enabled evaluation of gene-specific prognostic associations while minimizing stromal-related bias. Hazard ratio–adjusted and stroma-adjusted *P* values are provided. We also utilized ssGSEA to categorize PDAC patients from the TCGA dataset based on their level of expression of the matrisome genes of the CD8^hi^ or CD8^lo^ signatures. Gene set score was examined for its effect on survival by separating patients into high/low-score subgroups based on quantile dichotomization, with survival differences assessed via Cox models with stromal fraction as a covariate. The R packages used in this analysis included survminer, survival, and GSVA.

### Data visualization and statistics

**Figure 1.**
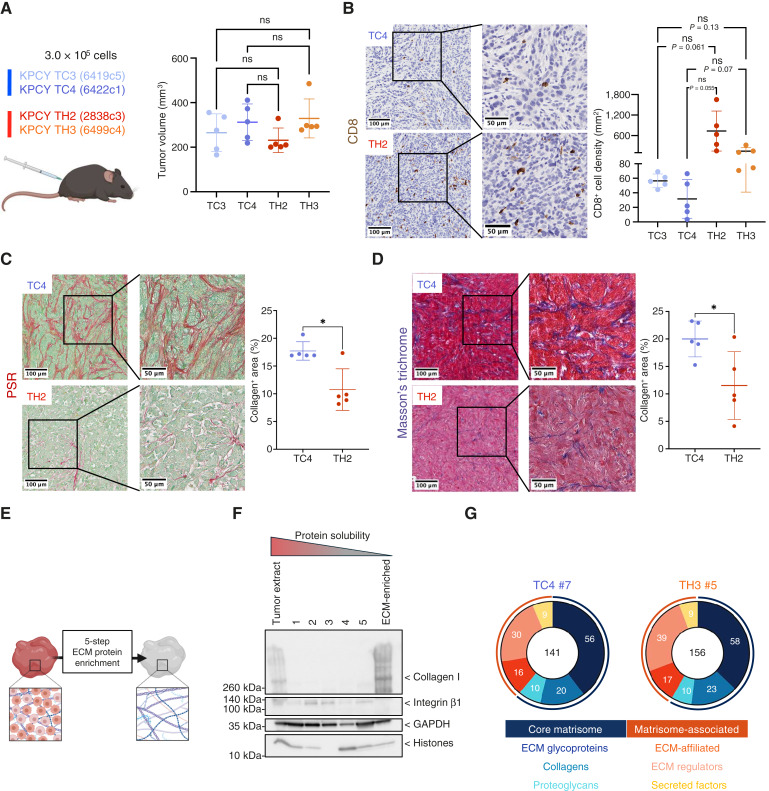
Experimental design and characterization of the ECM of the model system. **A,** Left, Schematic representation of experimental design and cell lines used. Right, Dot plot represents the tumor volumes of each sample (*n* = 5/group) at experimental endpoint. The average tumor volume for each tumor group is indicated by a bar, and the error bars represent the standard deviation (SD). Statistical analysis was performed using the Welch *t* test, assuming unequal variance, and statistical significance is depicted as follows: ns, not significant. **B,** Two sections of each tumor sample (*n* = 5/group) were stained using an anti-CD8 antibody. Representative images of CD8 staining of CD8^lo^ (TC4) and CD8^hi^ (TH2) tumors are shown (scale bar, 100 μm). Higher-magnification insets are also provided (scale bar, 50 μm). Dot plot represents the density of CD8^+^ T cells per tumor area (average of two sections from each tumor); the average value for each tumor group is indicated by a bar, and the error bars represent the SD. Statistical analysis was performed using the Welch *t* test, assuming unequal variance, and statistical significance is depicted as follows: ns, not significant. **C,** Two sections of each tumor sample (*n* = 5/group) were stained using Picrosirius red (PSR) to assess fibrillar collagen content. Representative images of PSR staining of CD8^lo^ (TC4) and CD8^hi^ (TH2) tumors are shown (scale bar, 100 μm). Higher-magnification insets are also provided (scale bar, 50 μm). Dot plot represents the proportion of PSR-positive area (i.e., “collagen-positive” area). The average positivity for each tumor group is indicated by a bar, and the error bars represent the SD. Statistical analysis was performed using the Welch *t* test, assuming unequal variance, and statistical significance is depicted as follows: *, *P* < 0.05. **D,** Two sections of each tumor sample (*n* = 5/group) were stained using Masson’s trichrome to assess fibrillar collagen content. Representative images of Masson’s trichrome staining of CD8^lo^ (TC4) and CD8^hi^ (TH2) tumors are shown (scale bar, 100 μm). Higher-magnification insets are also provided (scale bar, 50 μm). Dot plot represents the proportion of Masson’s trichrome–positive area (i.e., “collagen-positive” area). The average positivity for each tumor group is indicated by a bar, and the error bars represent the SD. Statistical analysis was performed using the Welch *t* test, assuming unequal variance, and statistical significance is depicted as follows: *, *P* < 0.05. **E,** Schematic depiction of the experimental workflow leading to the enrichment of ECM proteins from tumors. **F,** Representative immunoblots illustrate the sequential extraction of soluble non-ECM proteins (integrin β1, GAPDH, and histones) and enrichment of ECM proteins (collagen I) in the “ECM-enriched” protein fraction. **G,** Donut charts illustrate the distribution of matrisome proteins identified by MS across matrisome categories in representative samples of CD8^lo^ (sample TC4#7) and CD8^hi^ (sample TH3#5) tumors. Core matrisome proteins include ECM glycoproteins (dark blue), collagens (light blue), and proteoglycans (teal); matrisome-associated proteins include ECM-affiliated proteins (dark orange), ECM regulators, including ECM-remodeling enzymes and their inhibitors (light orange), and secreted factors, including ECM-bound growth factors and cytokines (yellow). [**A,** Created in BioRender. Naba, A. (2026) https://BioRender.com/gvbo9zk; **E,** Created in BioRender. Naba, A. (2026) https://BioRender.com/k8ocagd.]

All statistical analyses were performed using PRISM (GraphPad). The statistical test used and significance is mentioned in respective figure legends. All graphs were plotted using PRISM (GraphPad). Proportional Venn diagrams were generated using BioVenn: https://www.biovenn.nl/ (56). The intersection of multiple datasets was visualized using UpSetR and the online application: https://gehlenborglab.shinyapps.io/upsetr/ (57). Heat maps were generated using Morpheus: https://software.broadinstitute.org/morpheus. Panels in Fig. 1A and E were generated using BioRender: https://www.biorender.com/.

## Results

### Characterization of the stromal composition of CD8^lo^ and CD8^hi^ subcutaneous KPCY PDAC

To study the possible correlation between immune cell infiltration and ECM composition, we used a panel of previously established and characterized PDAC cell lines derived from a genetically engineered mouse model expressing Kras^LSL-G12D/+^; Trp53^LSL-R172H/+^; Pdx1-Cre; Rosa26^YFP/YFP^, (referred to as KPCY PDAC; ref. 37). We selected to work with two cell lines, KPCY TC3 (6419c5) and KPCY TC4 (6422c1), that when injected in mice form tumors with low CD8^+^ lymphocyte infiltration (further termed, CD8^lo^ or “cold” tumors) and with two cell lines, KPCY TH2 (2838c3) and KPCY TH3 (6499c4), that when injected in mice form tumors with higher CD8^+^ lymphocyte infiltration (further termed, CD8^hi^ or “hot” tumors; Fig. 1A). Importantly, and as previously reported (37), we confirmed that the cell lines retained their phenotype when injected subcutaneously [Fig. 1B (left)] and present trending differences in the level of CD8^+^ lymphocyte infiltration [Fig. 1B (right)]. We also observed broader microenvironmental changes with CD8^lo^ tumors characterized by a smaller number of αSMA^+^ CAFs (Supplementary Fig. S2A), a trend toward less CD4^+^ T-cell infiltration (Supplementary Fig. S2B; *P* = 0.0718), in line with the observations published in the article reporting the development of this model system, and a statistically significant higher levels of F4/80^+^ macrophages (Supplementary Fig. S2C) and Ly6G^+^ neutrophils (Supplementary Fig. S2D).

Importantly, we found that CD8^lo^ tumors have a statistically significantly higher ECM content than CD8^hi^ tumors based on two histologic stains specific for fibrillar collagens, Picrosirius red (Fig. 1C; *P* = 0.0104), and Masson’s trichrome (Fig. 1D; *P* = 0.0343). The observation that CD8^lo^ tumors have higher fibrillar collagen content and yet less SMA^+^ cells, often viewed as the main producer of collagens in the TME, suggests that either the CAFs in CD8^lo^ tumors produce more fibrillar collagens, that other cell populations within the CD8^lo^ TME produce fibrillar collagens, or perhaps that the collagen ECM of the CD8^lo^ TME is more stable and less remodeled.

### Proteomics identifies signatures correlating with CD8 lymphocyte infiltration

**Figure 2.**
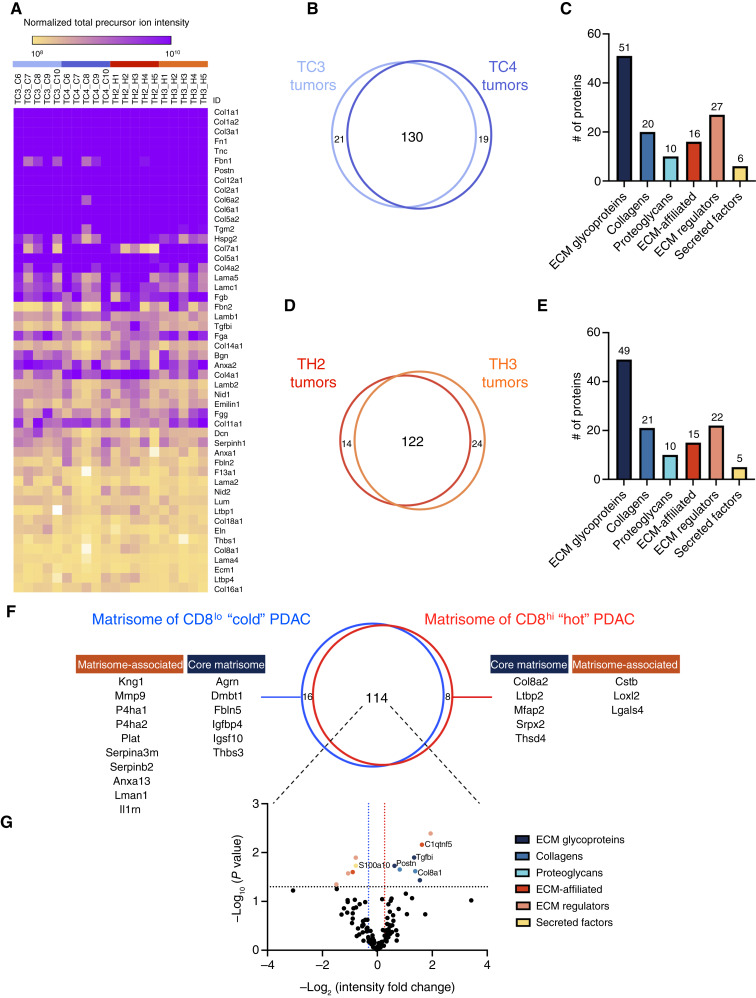
Proteomic definition of the matrisome of CD8^lo^ and CD8^hi^ KPCY tumors. **A,** Heat map depicts the abundance of the top 50 matrisome proteins across the 20 tumors profiled, inferred using total precursor ion intensities; related to Supplementary Table S2C. **B,** Proportional Venn diagram represents the overlap between the matrisome proteins detected in at least three of the five independent biological replicates of the TC3 (light blue) and TC4 (dark blue) CD8^lo^ KPCY tumors. The 130 matrisome proteins detected in both tumor groups based on these stringent inclusion criteria constitute the matrisome of CD8^lo^ tumors; related to Supplementary Table S2D. **C,** Bar graph represents the distribution of the 130 matrisome proteins detected in both tumor groups across the different matrisome categories. **D,** Proportional Venn diagram represents the overlap between the matrisome proteins detected in at least three of the five biological replicates of the TH2 (red) and TH3 (orange) CD8^hi^ KPCY tumors. The 122 matrisome proteins detected in both tumor groups based on these stringent inclusion criteria constitute the matrisome of CD8^hi^ tumors; related to Supplementary Table S2D. **E,** Bar graph represents the distribution of the 122 matrisome proteins detected in both tumor groups across the different matrisome categories. **F,** Proportional Venn diagram represents the overlap between the matrisome of CD8^lo^ (blue) and CD8^hi^ (red) tumors defined above (see Fig. 2B and D, respectively); related to Supplementary Table S2D. **G,** Volcano plot represents the change in normalized total precursor ion intensity (i.e., protein abundance) of the 114 matrisome proteins detected in both CD8^lo^ (*n* = 10) and CD8^hi^ (*n* = 10). Proteins detected in statistically differential abundance (Student *t* test *P* < 0.05 and ±20% change in abundance) are depicted in the upper left and right quadrants and color-coded based on the matrisome category they belong to.

To determine the protein composition of the ECM of CD8^lo^ and CD8^hi^ tumors, we first decellularized the tumors to achieve an enrichment of ECM proteins (Fig. 1E). Using immunoblotting, we confirmed the extraction of intracellular proteins over the course of the five-step decellularization process and the resulting enrichment of ECM components (Fig. 1F). ECM-enriched samples were subjected to proteolytic digestion, and the resulting peptides were fractionated using basic reversed-phase chromatography to achieve deep matrisome coverage and submitted to proteomic analysis using LC-MS/MS. We then used highly stringent criteria to accept peptide identification (<0.1% FDR) and protein identification (<1% FDR; detection with at least two unique peptides; Supplementary Table S2A). On average, we identified ∼150 distinct matrisome proteins in each tumor sample (Supplementary Table S2B) and found a consistent distribution of these proteins across the different categories of matrisome proteins, with ∼60% of the proteins belonging to core, structural, matrisome categories, including ECM glycoproteins, collagens, and proteoglycans, and ∼40% classified as matrisome-associated proteins (i.e., ECM-affiliated proteins, ECM regulators, including remodeling enzymes, and secreted factors, including ECM-bound growth factors or cytokines; Fig. 1G). Using total precursor ion intensity values, the gold-standard to infer protein abundance when performing label-free quantification proteomics, we found that the top five most abundant ECM proteins were the same between the two tumor groups: collagen I (Col1a1 and Col1a2), collagen III (Col3a1), fibronectin (Fn1), and tenascin C (Tnc; Fig. 2A; Supplementary Tables S2B and S2C). The fact that this subset of proteins is almost two orders of magnitude more abundant than any of the other matrisome proteins detected likely explains why we were unable to identify differences between hot and cold tumors, as we have observed by IHC.

We then defined the matrisome profile of each tumor model (TC3, TC4, TH2, and TH3) as the ensemble of proteins detected in at least three of the five independent biological replicates of each tumor model (Supplementary Table S2D). Intragroup comparison revealed a remarkable overlap between each biological replicate within each tumor model (Supplementary Fig. S3), validating the robustness of our experimental pipeline. Similarly, intergroup comparison revealed a large overlap (130 proteins) between the matrisome profiles of the two CD8^lo^ tumor models (i.e., TC3 vs. TC4; Fig. 2B and C). A similar proportion was observed when comparing the matrisome profiles of the two CD8^hi^ tumor models (i.e., TH2 vs. TH3; Fig. 2D and E). Of note, the matrisome profiles of CD8^lo^ and CD8^hi^ tumors presented a similar distribution across matrisome categories, with the largest number of proteins belonging to the ECM glycoprotein, collagen, and ECM regulator categories (Fig. 2C and E, respectively).

Last, we defined the matrisome profile of CD8^lo^ tumors and CD8^hi^ tumors as the ensemble of proteins detected in at least three of the five independent biological replicates and in both tumor models for a given tumor type. The comparison of these profiles identified 114 matrisome proteins detected in both tumor types (Fig. 2F). Importantly, we identified a set of 16 matrisome proteins characteristic of CD8^lo^ tumors [Fig. 2F (left)]. This set included six core matrisome components: agrin (Agrn), Dmbt1, fibulin 5 (Fbln5), insulin growth factor binding protein 4 (Igfbp4), Igsf10, and thrombospondin 3 (Thbs3) and 10 matrisome-associated components, including the matrix metalloproteinase 9 (Mmp9), an enzyme known to degrade collagens, and, interestingly, two prolyl hydroxylases, P4ha1 and P4ha2. Prolyl hydroxylases are the enzymes responsible for the addition of hydroxyl groups on prolines in glycine–X–Y amino acid motifs, prominent in collagens, a posttranslational modification contributing to the stability of the collagen triple helix and hence of the entire collagen meshwork within the ECM (58). The observation that both enzymes are detected in higher abundance in the ECM of CD8^lo^ tumors may contribute to explaining our early observation that these tumors have relatively higher fibrillar collagen content than CD8^hi^ tumors. It also suggests that collagen synthesis and stability may be higher than MMP9-mediated collagen degradation in cold tumors.

We also identified a set of eight matrisome proteins characteristic of CD8^hi^ tumors [Fig. 2F (right)]. This set comprised five core matrisome proteins and three matrisome-associated proteins, including the ECM crosslinking enzyme lysyl oxidase-like 2 (Loxl2), previously shown to associate with PDAC progression and the focus of active research and development efforts (59–62), demonstrating the rigor of our approach. It has previously been proposed that crosslinked, stiffer ECM hinders immune cell infiltration and function. Yet, we found that Loxl2 is present in higher abundance in CD8^hi^ tumors. These two somewhat opposite observations can be reconciled by our earlier observation that the fibrillar collagen content, substrate of Loxl2-mediated crosslinking, is lower in CD8^hi^ tumors, and so, rather than crosslinking, the enzyme-to-substrate ratio is the factor permitting or limiting factor to lymphocyte infiltration.

Quantitative analysis of the proteins detected in the matrisome of both tumor types was performed using normalized total precursor ion intensity [Supplementary Table S2B (columns F–Y)]. This allowed us to identify a set of proteins present in statistically significant higher abundance in CD8^lo^ [Fig. 2G (top left quadrant)], such as S100a10, and a set of proteins present in statistically significant higher abundance in CD8^hi^ tumors [Fig. 2G (top right quadrant)], such as periostin (Postn), the α1 chain of collagen VIII (Col8a1), or the TGFβ-induced protein (Tgfbi). Altogether, these results define a 21-matrisome-protein signature characteristic of CD8^lo^ tumors and a 15-matrisome-protein signature characteristic of CD8^hi^ tumors.

### Immunostaining assessment of the distribution of ECM proteins in CD8^lo^ and CD8^hi^ PDAC tumors

Next, we selected a subset of proteins detected with differential abundance between CD8^lo^ and CD8^hi^ tumors to assess their distribution patterns using IHC and support the proteomic results. We selected these proteins (fibulin 5 and MMP9 for cold PDACs; collagen VIII and TGFβ induced for hot PDACs) based on the availability of validated antibodies. In agreement with our proteomic data, fibulin 5 (Supplementary Fig. S4A) and MMP9 (Supplementary Fig. S4B) presented a distinctive extracellular distribution in the microenvironment of CD8^lo^ tumors but were not detected in CD8^hi^ tumors. Conversely, collagen VIII presented an extracellular distribution in the microenvironment of CD8^hi^ tumors but was not detected in CD8^lo^ tumors (Supplementary Fig. S4C). Interestingly, although we detected positive staining for the protein TGFβ induced in both CD8^lo^ and CD8^hi^ tumors (Supplementary Fig. S4D), the pattern was clearly intracellular in CD8^lo^ tumors and extracellular in CD8^hi^ tumors. We further confirmed this phenotype by assessing the presence of TGFβ induced by immunoblot and found that, although TGFβ induced was detected in protein samples extracted from CD8^lo^ tumors, it was enriched in a protein fraction of high solubility, so likely intracellular [Supplementary Fig. S4D (lower left)]. In contrast, in CD8^hi^ tumors, TGFβ induced was retained in the ECM-enriched protein fraction characterized by high insolubility [Supplementary Fig. S4D (lower right)]. Although performed on a small number of samples (*n* = 2 per tumor type), this qualitative analysis supports the potential of our discovery pipeline to identify ECM proteins of the TME.

### Signature analysis reveals different pathways activated in CD8^lo^ and CD8^hi^ PDAC tumors

**Figure 3.**
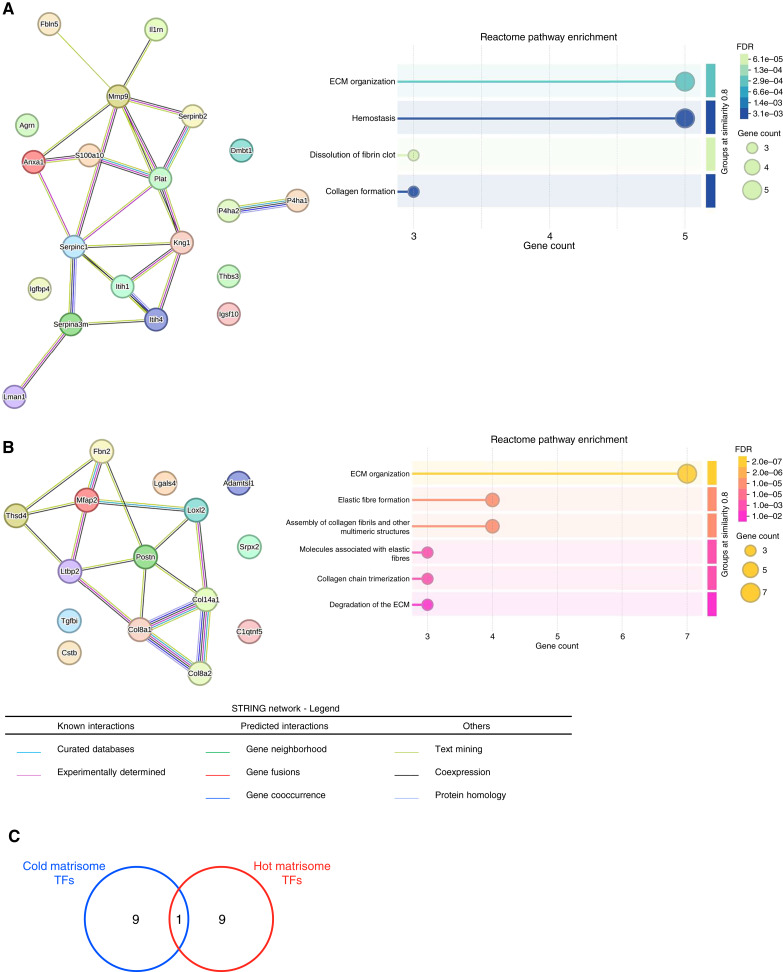
Signature analysis reveals different matrisome networks and pathways activated in CD8^lo^ and CD8^hi^ KPCY tumors. **A,** STRING analysis was applied to build a network connecting the proteins of the CD8^lo^ signature (left) and to identify Reactome pathways upregulated in CD8^lo^ tumors (right). **B,** STRING analysis was applied to build a network connecting the proteins of the CD8^hi^ signature (left) and to identify Reactome pathways upregulated in CD8^hi^ tumors (right). **C,** Proportional Venn diagram represents the overlap between the top 10 TFs upstream of the genes encoding the matrisome proteins of the CD8^lo^ signature (blue circle; see also Table 1) or encoding the matrisome proteins of the CD8^hi^ signature (red circle; see also Table 2).

Protein–protein interactions (PPI) between matrisome components intrinsically determine their functions. Indeed, PPIs allow the assembly of the complexes forming the fibers of the ECM scaffold, whereas interactions between ECM-remodeling enzymes and their substrates are critical for ECM remodeling. We thus thought to determine whether the components of the matrisome signatures identified in this study participated in similar protein networks using STRING analysis (48). Distinct nodes were identified for the CD8^lo^ and CD8^hi^ matrisome signatures [Fig. 3A and B (left), respectively]. However, it is important to note the complete absence of connection between many components, while they all belong to the same compartment. This demonstrates the critical gap in knowledge that remains around the ECM and highlights the importance of descriptive studies establishing phenotypic correlations that can then enrich the list of signatures in pathway databases (63).

Next, we thought to determine whether the components of the matrisome signatures identified participated in common signaling pathways. As expected, and due to our experimental design, both signatures were enriched for genes involved in “ECM organization” as defined by the Reactome database (49). In addition, the CD8^lo^ (i.e., cold tumor) matrisome signature was enriched for genes involved in pathways modulating collagen formation, in line with the phenotype reported in Fig. 1C and D, hemostasis, and dissolution of fibrin clot [Fig. 3A (right)], suggestive of a higher vascularization. However, quantification of the vasculature of hot and cold tumors did not reveal any difference (Supplementary Fig. S2E; *P* = 0.5932). An alternative hypothesis is that CD8^lo^ tumors present a leakier vasculature, which can explain the presence of circulating proteins sequestered in the insoluble tumor ECM. In contrast, the CD8^hi^ (i.e., hot tumor) matrisome signature was enriched for genes playing roles in pathways modulating elastic fiber formation or degradation of the ECM [Fig. 3B (right)]. Further examination of the ECM signature of CD8^hi^ tumors revealed that several of its components are part of the TGFβ signaling pathway, such as the latent TGFβ-binding protein 2, TGFβ induced, and fibrillin 2. It is tempting to speculate that a microenvironment enriched in elastic components is more permissive to immune cell infiltration than a microenvironment rich in fibrillar collagen stabilized by extensive posttranslational modifications, as suggested by the ECM protein signature of cold tumors.

Last, we sought to determine whether specific pathways could predict the expression of the genes of the CD8^lo^ and CD8^hi^ ECM signatures. To do so, we performed a TF enrichment analysis using the ChIP-X Enrichment Analysis 3 (ChEA3), a web-based tool that ranks TFs associated with user-submitted gene sets (50). We found that vastly different arrays of TFs governed the expression of the ECM genes of the CD8^lo^ and CD8^hi^ signatures, with the TFs ATF5, TIGD2, and CREB3L1 significantly enriched in the CD8^lo^ signature (Table 1) and FOXC2, PRRX2, and FOXS1 enriched in the CD8^hi^ signature (Table 2). The comparison of the top 10 TFs governing the expression of the genes encoding the matrisome proteins of the CD8^lo^ signature or encoding the matrisome proteins of the CD8^hi^ signature reveals that only one TF, OSR1 (odd-skipped related 1), a zinc-finger TF, controlled subsets of genes part of the CD8^lo^ and CD8^hi^ matrisome signatures (Fig. 3C), indicating that different transcriptional are at play in both tumor and stromal cells to regulate the expression of matrisome genes correlating with CD8 T-cell infiltration. Whereas the majority of the TFs identified in this analysis have not been previously associated with lymphocyte biology or the ECM, EGR2 (early growth response 2), found to regulate six genes of the matrisome signature characteristic of CD8^lo^ tumors (Table 1), has been associated with different aspect of T-cell functions (64–67) and ECM remodeling and fibrosis, in line with our observation that CD8^lo^ tumors have higher collagen content (68–70). Interestingly, and in agreement with our observations, a recent study linked CREB3L1 (cAMP responsive element binding protein 3 like 1), found to regulate six genes of the matrisome signature characteristic of CD8^lo^ tumors, to PDAC progression and resistance to immunotherapy (71). Last, few TFs governing the expression of genes of both the CD8^lo^ (e.g., CREB3L1 and SNAI2) and CD8^hi^ (e.g., FOXC2 and PRRX2) matrisome signatures have been associated with epithelial-to-mesenchymal transition, a program in part associated with increased ECM production and remodeling (72, 73), or the development of ECM-rich structures such as bones, cartilage, and teeth. In the future, it would be interesting to determine whether these TFs and the pathways that activate them predict immune cell infiltration or response to therapy.

**Table 1.** Top 10 TFs governing the expression of the genes of the ECM signature characteristic of CD8^lo^ tumors.

Rank	TF	Score	# Overlapping genes	Overlapping genes
1	ATF5	6.143E−04	6	*ITIH4*, *IL1RN*, *SERPINC1*, *ITIH1*, *MMP9*, and* KNG1*
2	TIGD2	6.223E−04	5	*ITIH4*, *LMAN1*, *SERPINC1*, *ITIH1*, and* KNG1*
3	CREB3L1	7.123E−04	6	*ANXA1*, *IGFBP4*, *P4HA2*, *PLAT*, *FBLN5*, and *S100A10*
4	OSR1	0.001229	4	*IGFBP4*, *P4HA2*, *FBLN5*, and *THBS3*
5	AR	0.001245	4	*ITIH4*, *SERPINC1*, *ITIH1*, and* KNG1*
6	EGR2	0.001425	6	*IL1RN*, *ANXA1*, *IGFBP4*, *PLAT*, *MMP9*, and* S100A10*
7	SNAI2	0.001843	4	*LMAN1*, *IGFBP4*, *P4HA2*, and* FBLN5*
8	CUX2	0.001867	4	*ITIH4*, *SERPINC1*, *ITIH1*, and* KNG1*
9	FOSB	0.002137	6	*ANXA1*, *SERPINB2*, *IGFBP4*, *PLAT*, *MMP9*, and* S100A10*
10	PROX1	0.002457	4	*ITIH4*, *SERPINC1*, *ITIH1*, and* KNG1*

**Table 2.** Top 10 TFs governing the expression of the genes of the ECM signature characteristic of CD8^hi^ tumors.

Rank	TF	Score	# Overlapping genes	Overlapping genes
1	FOXC2	6.143E−04	8	*ADAMTSL1*, *POSTN*, *SRPX2*, *COL8A1*, *LTBP2*, *TGFBI*, *THSD4*, and *LOXL2*
2	PRRX2	6.223E−04	6	*COL14A1*, *MFAP2*, *COL8A2*, *COL8A1*, *LTBP2*, and *LOXL2*
3	FOXS1	7.123E−04	9	*CSTB*, *POSTN*, *SRPX2*, *COL14A1*, *MFAP2*, *COL8A1*, *TGFBI*, *FBLN2*, and *LOXL2*
4	OSR1	0.001229	7	*ADAMTSL1*, *COL14A1*, *LTBP2*, *TGFBI*, *FBLN2*, *THSD4*, and *LOXL2*
5	AEBP1	0.001245	6	*COL14A1*, *C1QTNF5*, *MFAP2*, *COL8A2*, *COL8A1*, and* LTBP2*
6	PRRX1	0.001843	7	*ADAMTSL1*, *POSTN*, *COL14A1*, *LTBP2*, *TGFBI*, *FBLN2*, and *LOXL2*
7	MKX	0.001867	5	*ADAMTSL1*, *COL14A1*, *MFAP2*, *COL8A2*, and *COL8A1*
8	BNC2	0.002137	7	*ADAMTSL1*, *POSTN*, *COL14A1*, *COL8A1*, *TGFBI*, *FBLN2*, and* LOXL2*
9	GLIS1	0.002457	7	*ADAMTSL1*, *COL8A2*, *COL8A1*, *LTBP2*, *TGFBI*, *FBLN2*, and *LOXL2*
10	DUXA	0.002489	5	*COL14A1*, *C1QTNF5*, *COL8A2*, *COL8A1*, and *LTBP2*

### Multiple cell populations of the TME contribute to the human PDAC matrisome

**Figure 4.**
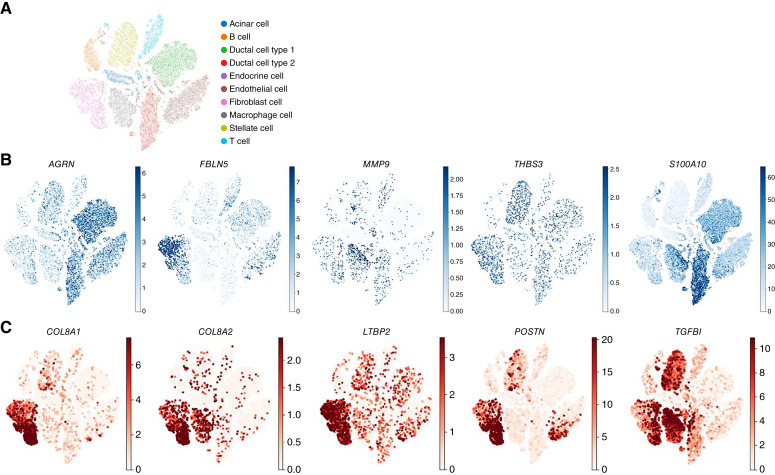
Identification of the cell types producing matrisome proteins in human PDAC microenvironments. **A,** t-distributed stochastic neighbor embedding (t-SNE) represents the distribution of the different cell populations in human PDAC samples. **B,** t-SNE of five genes *AGRN*, *FBLN5*, *MMP9*, *THBS3*, and *S100A10*, encoding proteins detected in higher abundance in CD8^lo^ tumors, demonstrates the contribution of different cell populations to the matrisome of these tumors. **C,** t-SNE of five genes, *COL8A1*, *COL8A2*, *LTBP2*, *POSTN*, and *TGFBI*, encoding proteins detected in higher abundance in CD8^hi^ tumors, demonstrates the contribution of different cell populations to the matrisome of these tumors.

ECM proteins are synthesized intracellularly and secreted into the extracellular space by multiple cell types. The assembled ECM of the TME is thus the product of multiple cell types. Whereas fibroblasts and cells of mesenchymal origins, such as stellate cells in the pancreas, are the main producers of core matrisome components, many other cell types can contribute to the production of the tumor ECM. We thus sought to determine which cell populations within human PDAC express genes of the cold and hot matrisome signatures. To do so, we leveraged a previously published scRNA-seq dataset (51) reporting gene expression levels across 10 cell populations found in PDAC (Fig. 4A). As expected, we found that genes encoding the proteins of the CD8^lo^ (cold) and CD8^hi^ (hot) matrisome signatures were expressed by different cell populations within the human PDAC microenvironment. For example, for the genes encoding proteins associated with the low CD8^+^ T-cell infiltration, *AGRN* is expressed by both ductal cell types 1 and 2 and endothelial cells, *FBLN5* is expressed by fibroblasts and endothelial cells, *MMP9* is expressed by macrophages, fibroblasts, and ductal cell type 2, *THBS3* is expressed by stellate cells and fibroblasts, and *S100A10 *is expressed by ductal cell types 1 and 2 and a subset of macrophages (Fig. 4B). Similarly, for the genes encoding proteins associated with a higher CD8^+^ T-cell infiltration, *COL8A1*, *COL8A2*, *LTBP2*, *POSTN*, and *TGFBI* are primarily expressed by CAFs. In addition, *LTBP2* and *POSTN* are also expressed by stellate cells and endothelial cells, whereas TGFBI is expressed by stellate cells, macrophages, and a subset of ductal cell type 2 (Fig. 4C). Although gene expression does not automatically equal protein production, these selected examples demonstrate that multiple cell populations within the human PDAC microenvironment likely contribute to the production of the components of the tumor ECM.

### Matrisome gene signatures correlate with CD8^+^ T-cell infiltration in human PDAC

**Figure 5.**
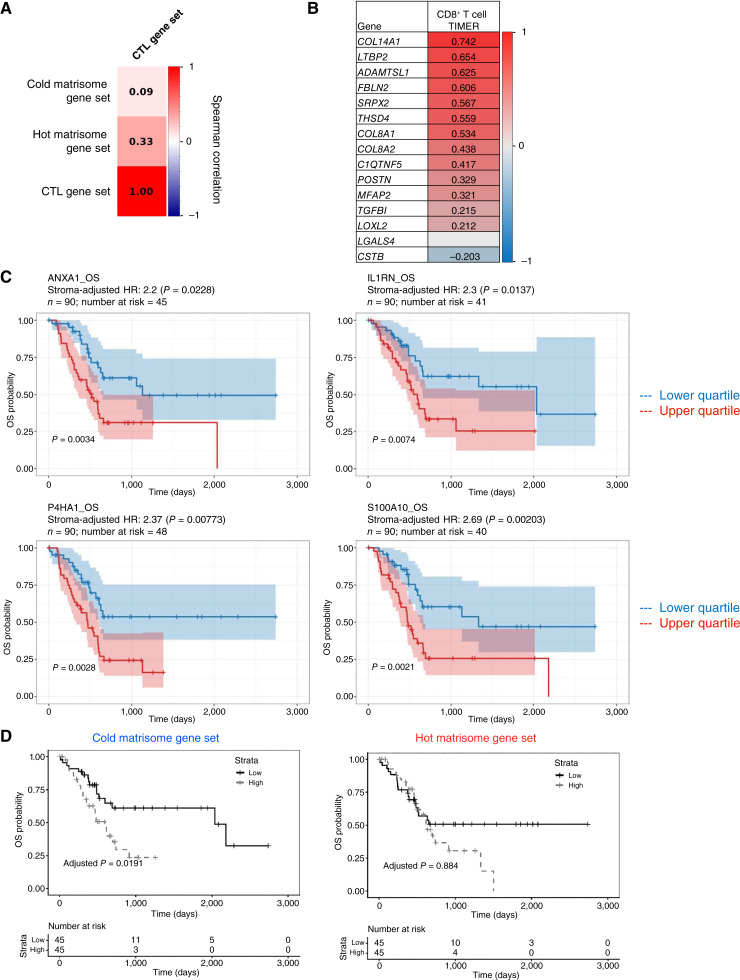
Prognostic value of the expression of matrisome genes on CD8^+^ T-cell infiltration of human PDACs and PDAC patient survival. **A,** Pairwise Spearman correlation coefficients were calculated between the cold ECM gene set or the hot matrisome gene set and a CTL gene set. Color scale reflects the strength and direction of correlation, with red indicating a positive correlation and blue indicating a negative correlation. **B,** Heat map represents the association between the expression of the genes from the hot PDAC matrisome signatures with CD8^+^ T-cell infiltration assessed using the TIMER2.0 algorithm in the TCGA PDAC RNA-seq dataset. A positive association score is indicated in red, whereas a negative association is indicated in blue. Associations with a *P* value less than 0.05 were considered statistically significant. **C,** Kaplan–Meier plots represent the survival probability (*y*-axis) over time (*x*-axis) of patients with PDAC (*n* = 90) stratified based on high (upper quartile; black line) or low (lower quartile; gray line) expression levels of the following matrisome genes encoding matrisome proteins characteristic of CD8^lo^ tumors: *ANXA1*, *IL1RN*, *P4HA1*, and *S100A10*. Statistical analysis was performed using the Cox proportional hazard model, and corresponding adjusted *P* value and stroma-adjusted hazard ratios (HR) and *P* values are indicated. **D,** Kaplan–Meier plots represent the OS probability of patients with PDAC (*n* = 90) stratified into two groups based on high expression (upper quartile; black line) or low expression (lower quartile; gray line) scores of the CD8^lo^ matrisome signature (left) or CD8^hi^ matrisome signature (right) tumors. Statistical analysis was performed using the Cox proportional hazard model, and corresponding HRs and stroma-adjusted significance (adjusted *P* value) are indicated.

To further explore the biological relevance of these ECM protein signatures, we examined their correlation with cytotoxic T lymphocyte (CTL) activity. We first performed ssGSEA to determine the degree to which the hot and cold matrisome gene sets correlated with a gene set indicative of CTL infiltration. We found that the hot matrisome gene set showed a moderate positive correlation with the CTL signature (r = 0.33). In contrast, the cold matrisome gene set exhibited a weaker correlation with the CTL signature (r = 0.09; Fig. 5A). We next applied the TIMER algorithm (53) to determine whether the expression of the genes encoding the ECM protein signatures defined in this study correlated with the abundance of different types of immune infiltrates in human PDAC patient samples of the TCGA PAAD cohort. We found that the expression of a subset of the genes of the CD8^hi^, hot, PDAC matrisome signature, including *COL14A1*, *ADAMTSL1*, *LTBP2*, *FBLN2*, and *THSD4*, positively correlated with the presence of CD8^+^ T-cell infiltrates in TCGA PDAC samples, inferred by the TIMER algorithm using defined subsets of biomarkers to annotate CD8^+^ T cells (e.g., TIMER, CIBERSORT, and EPIC; Fig. 5B; Supplementary Table S3A). Conversely, we found that the expression of a subset of the CD8^lo^, cold, PDAC matrisome signature, including *AGRN*, *P4HA1*, *P4HA2*, *IL1RN*, and *S100A10*, negatively correlated with CD8^+^ T-cell infiltrates (Supplementary Table S3B). The fact that there was not complete agreement between the expression of the genes of the gene sets derived from the proteomic signatures defined in this study and the level of immune cell infiltration can partly be attributed to the fact that there is a well-documented discrepancy between RNA expression level and protein abundance. It is also important to note that the data from TCGA include gene expression levels from all the cell types present in tumors, including tumor cells and stromal cells. It would be interesting in the future to correlate matrisome protein abundance in human PDAC samples and the level of immune cell content to further validate the relevance of the protein signature we identified in preclinical models of hot and cold PDACs.

### The cold matrisome gene signature correlates with patient outcome

Multiple studies across cancer types have reported a positive correlation between CD8^+^ T-cell infiltration and survival (28, 74, 75) or recurrence (76). We thus asked whether the level of expression of genes encoding the proteins of the cold tumor signature negatively correlated with patient survival. To do so, we retrieved expression data and clinical data from TCGA and plotted the survival probability of high versus low expressors (upper and lower quartiles) over time. We found that the level of expression of multiple genes of the cold matrisome signature, and particularly those found to negatively correlated with CD8^+^ T-cell infiltrates based on the TIMER analysis, namely* IL1RN*, *P4HA1*, and *S100A10*, and as *ANXA1*, associated with worse prognosis and OS (Fig. 5C; see *P* values), even when factoring in tumor purity as a covariate (see stroma-adjusted *P* values). Of note, the expression of the other genes did not correlate with survival. We also observed that patients expressing a higher level of genes in the entire “cold matrisome signature” had statistically significant worsen OS [Fig. 5D (left); *P*_adj_ = 0.0191]. However, we found no association between the level of expression of the “hot matrisome signature” and OS of patients with PDAC [Fig. 5D (right); *P*_adj_ = 0.884].

## Discussion

In this study, we used an ECM-focused proteomic pipeline to identify protein signatures correlating with lymphocyte infiltration in mouse models of PDACs and found that CD8^lo^ or “cold” tumors and CD8^hi^ or “hot” tumors exhibited different ECM compositions. We further showed that different populations within the TME express the genes encoding these proteins.

Previous studies have used histochemical or antibody-based stains to characterize the collagen content of PDACs. Using Elastica van Gieson staining of the PDAC ECM, Ashina and colleagues (32) found that, in a cohort of 169 patients with PDAC, lower collagen content associated with a poorer prognosis and shorter OS and that tumors with higher collagen content tended to have higher infiltration of CD8^+^ T cells, although this observation was not statistically significant (*P* = 0.09). However, in an independent study, Carstens and colleagues (31) found that neither the proportion of αSMA nor that of collagen I correlated with CD8^+^ T-cell infiltration. However, in this study we show that collagen I content anticorrelated with CD8^+^ T-cell density. Discrepancies between our observation and previously published studies can originate from the fact that we used tumors grown subcutaneously and that the skin environment is different from the pancreatic stroma in terms of cell populations and ECM composition. It would thus be interesting to further evaluate the correlation of CD8^+^ T-cell infiltration and collagen and ECM composition in larger panels of human samples and using multiple staining modalities to definitively conclude on whether or not the ECM can act as a physical barrier to immune cell recruitment.

αSMA^+^ CAFs are commonly thought to be the primary contributors to the production of a collagen-rich ECM within tumors (24, 77). Histologic analyses show that CD8^lo^ tumors have higher fibrillar collagen content but lower αSMA^+^ cell content. Few mechanisms can be proposed to explain this discrepancy: it is possible that not all CAFs were stained with the anti-αSMA antibody, and it would be thus interesting in the future to use additional fibroblast markers, such as the fibroblast-specific protein 1 (FSP1) or fibroblast activation protein (FAP) to have a more complete view of the fibroblastic population in the PDAC TME (77). It is also possible that CAFs associated with CD8^lo^ tumors produce more fibrillar collagens; other cell populations within the CD8^lo^ TME can also contribute to the production of fibrillar collagens. Lastly, the collagen ECM in the CD8^lo^ TME could be more stable. Our proteomic data, showing a higher abundance of enzymes (P4ha1 and P4ha2) involved in the hydroxylation of prolines of collagens and the stabilization of triple-helical collagens in the biosynthetic pathway (58), provides support for the third hypothesis. Interestingly, Peng and colleagues have shown that, in melanoma, collagen promotes resistance to immune checkpoint inhibitors (ICI) and CD8^+^ T-cell exhaustion. They further show that decreasing collagen stiffness by inhibiting the collagen crosslinking enzyme lysyl oxidase-like 2 (LOXL2) resulted in increased CD8^+^ T-cell infiltration and tumors gained sensitivity to ICIs (78). However, in our dataset, Loxl2 was detected in greater abundance in hot tumors, suggesting that different aspects of collagen remodeling, stabilization via hydroxylation versus crosslinking, may be at play in different cancer types. Nonetheless, it is tempting to speculate that modulating the collagen content in the PDAC TME might alter a tumor’s phenotype and favor immune cell infiltration and activity.

Due to their extracellular localization and presence in high abundance, ECM proteins are appealing candidates to serve as anchors for the targeted delivery of therapeutic payloads to specific sites such as primary tumors. Indeed, approaches designed to use collagen or tenascin C to anchor and concentrate cytokines or ICIs to the tumor ECM have shown promise in animal models (79–83). We thus propose that the proteins of the matrisome signature characteristic of CD8^hi^ tumors defined here could serve as specific anchors to deliver to tumors ICIs, as these tumors should readily be able to respond to this modality.

Our findings also underscore the functional relevance of matrisome composition in shaping the immune landscape of tumors. The positive correlation between the hot PDAC matrisome signature, along with enriched expression of markers of CD8^+^ T cells indicative of CTL activity, suggests that these ECM components may actively support T-cell infiltration, survival, and effector function. In contrast, the cold matrisome signature is largely absent in T cell–rich tumor environments, pointing to a role in immune exclusion or suppression. This dichotomy highlights the potential of matrisome profiles not only as biomarkers of immune contexture but also as modulators of antitumor immunity.

The emerging concept of “matritherapy” proposes the development of strategies to modulate either the structure, composition, or signaling functions of the ECM to achieve therapeutic benefits (36). For example, in a recent study, Wu and colleagues (84) showed that knocking down the expression of the ECM protein fibronectin or preventing the interaction of fibronectin with its receptors, α5β1 and αvβ3 integrins, significantly decreased the growth of subcutaneous PDAC in mice. Targeting specific matrisome components may thus offer novel therapeutic strategies to reprogram the TME and enhance immune responsiveness, particularly in tumors characterized by a cold, immune-desert phenotype.

## Supplementary Material

Supplementary Figure 1 Quantification of CD8+ T-cell infiltration using HALO

Supplementary Figure 2 Characterization of the stroma and immune landscape of CD8lo and CD8hi tumors

Supplementary Figure 3 Intragroup reproducibility of the proteomic output

Supplementary Figure 4 Qualitative assessment of the distribution of proteins detected in differential abundance between CD8hi and CD8lo KPCY tumors using IHC

Supplementary Table 1 Antibody list

Supplementary Table 2 Complete proteomic dataset

Supplementary Table 3 Tumor IMmune Estimation Resource (TIMER) analysis

## Data Availability

Raw MS data and accompanying metadata file in the Sample and Data Relationship Format (85) have been deposited to the ProteomeXchange Consortium (86) via the PRIDE partner repository (87) with the dataset identifier PXD060932. Representative full-section scans of tumors stained for ECM proteins of interest were deposited in Figshare and are available at https://doi.org/10.6084/m9.figshare.30687044. All other data are available upon request to the corresponding author.
