# Supplementary material for: Identification of Pancreatic Ductal Adenocarcinoma Extracellular Matrix Signatures from In-Depth Proteomic Profiling that Correlate with Lymphocyte Infiltration
Source: Cancer Res Commun. 2026 Jun 5;6(6):1319–35. doi: 10.1158/2767-9764.CRC-25-0460 (PMC13236633; doi:10.1158/2767-9764.CRC-25-0460)
Supplement: Supplementary Figure 1 — Quantification of CD8+ T-cell infiltration using HALO [file crc-25-0460_supplementary_figure_1_suppsf1.pdf]

Supplementary Figure 1. Quantification of CD8$^+$ T-cell infiltration using HALO

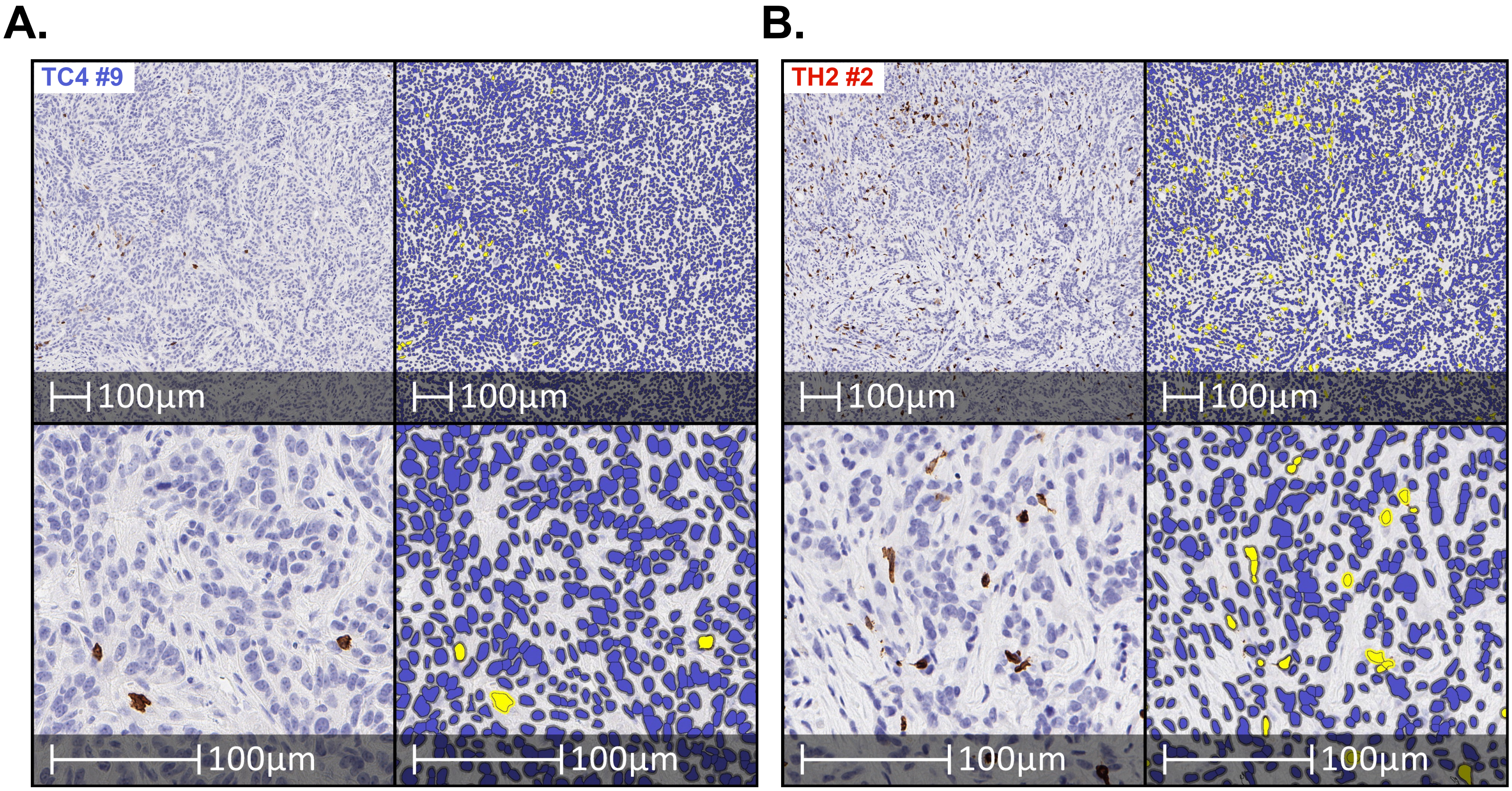

Supplementary Figure 1. Quantification of CD8$^+$ T-cell infiltration using HALO; *Related to Figure 1*

A. Representative images showing CD8 staining (brown) of CD8^{lo} tumor section (TC4 #9) at different magnifications and respective masks (right panel) after adjusting threshold to quantify CD8$^+$ T cells (yellow) and CD8$^-$ cells (blue) using the multiplex IHC algorithm of the HALO software.

B. Representative images showing CD8 staining (brown) of CD8^{hi} tumor section (TH2 #2) at different magnifications and respective masks (right panel) after adjusting threshold to quantify CD8$^+$ T cells (yellow) and CD8$^-$ cells (blue) using the multiplex IHC algorithm of the HALO software.
