# Supplementary material for: Identification of Pancreatic Ductal Adenocarcinoma Extracellular Matrix Signatures from In-Depth Proteomic Profiling that Correlate with Lymphocyte Infiltration
Source: Cancer Res Commun. 2026 Jun 5;6(6):1319–35. doi: 10.1158/2767-9764.CRC-25-0460 (PMC13236633; doi:10.1158/2767-9764.CRC-25-0460)
Supplement: Supplementary Figure 2 — Characterization of the stroma and immune landscape of CD8lo and CD8hi tumors [file crc-25-0460_supplementary_figure_2_suppsf2.pdf]

Supplementary Figure 2. Characterization of the stroma and immune landscape of CD8^{lo} and CD8^{hi} tumors

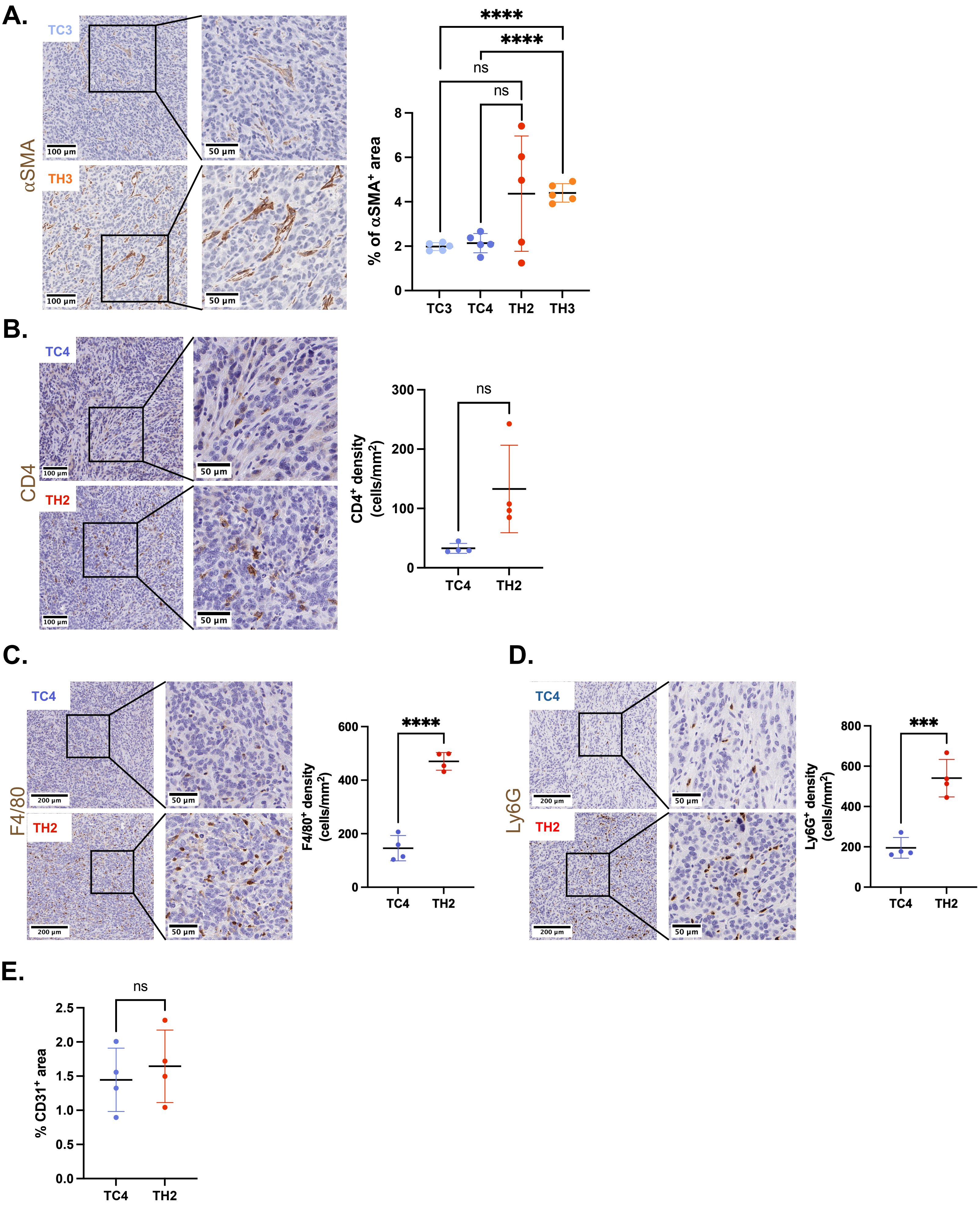

Supplementary Figure 2. Characterization of the stroma and immune landscape of $CD8^{lo}$ and $CD8^{hi}$ tumors; *Related to Figure 1*

A. Two sections of each tumor sample (n=5/group) were stained using an anti-αSMA antibody. Representative images of αSMA staining of $CD8^{lo}$ (TC3) and $CD8^{hi}$ (TH3) tumors are shown (scale bar: 100μm). Higher-magnification insets are also provided (scale bar: 50μm). Dot plot represents the proportion of αSMA-positive tumor area. The average value for each tumor group is indicated by a bar and the error bars represent the standard deviation (SD). Statistical analysis was performed using the Welch's t-test, assuming unequal variance, and statistical significance is depicted as follows: ns-not significant; ****p <0.0001.

B. One section of each tumor sample (n=4/group) was stained using an anti-CD4 antibody. Representative images of CD4 staining of $CD8^{lo}$ (TC4) and $CD8^{hi}$ (TH2) tumors are shown (scale bar: 100μm). Higher-magnification insets are also provided (scale bar: 50μm). Dot plot represents the density of $CD4^{+}$ T cells per tumor area; the average value for each tumor group is indicated by a bar and the error bars represent the standard deviation (SD). Statistical analysis was performed using the Welch's t-test, assuming unequal variance, and statistical significance is depicted as follows: ns-not significant.

C. One section of each tumor sample (n=4/group) was stained using an anti-F4/80 antibody. Representative images of F4/80 staining of $CD8^{lo}$ (TC4) and $CD8^{hi}$ (TH2) tumors are shown (scale bar: 200μm). Higher-magnification insets are also provided (scale bar: 50μm). Dot plot represents the density of $F4/80^{+}$ cells per tumor area; the average value for each tumor group is indicated by a bar and the error bars represent the standard deviation (SD). Statistical analysis was performed using the Welch's *t*-test, assuming unequal variance, and statistical significance is depicted as follows: *** p <0.0001.

D. One section of each tumor sample (n=4/group) was stained using an anti-Ly6G antibody. Representative images of Ly6G staining of $CD8^{lo}$ (TC4) and $CD8^{hi}$ (TH2) tumors are shown (scale bar: 200μm). Higher-magnification insets are also provided (scale bar: 50μm). Dot plot represents the density of $Ly6G^{+}$ cells per tumor area; the average value for each tumor group is indicated by a bar and the error bars represent the standard deviation (SD). Statistical analysis was performed using the Welch's *t*-test, assuming unequal variance, and statistical significance is depicted as follows: *** p <0.0001.

E. One section of each tumor sample (n=4/group) was stained using an anti-CD31 antibody. Dot plot represents the proportion of CD31-positive tumor area. The average value for each tumor group is indicated by a bar and the error bars represent the standard deviation (SD). Statistical analysis was performed using the Welch's t-test, assuming unequal variance, and statistical significance is depicted as follows: ns-not significant.
