# Supplementary material for: Identification of Pancreatic Ductal Adenocarcinoma Extracellular Matrix Signatures from In-Depth Proteomic Profiling that Correlate with Lymphocyte Infiltration
Source: Cancer Res Commun. 2026 Jun 5;6(6):1319–35. doi: 10.1158/2767-9764.CRC-25-0460 (PMC13236633; doi:10.1158/2767-9764.CRC-25-0460)
Supplement: Supplementary Figure 3 — Intragroup reproducibility of the proteomic output [file crc-25-0460_supplementary_figure_3_suppsf3.pdf]

Supplementary Figure 3. Intragroup reproducibility of the proteomic output

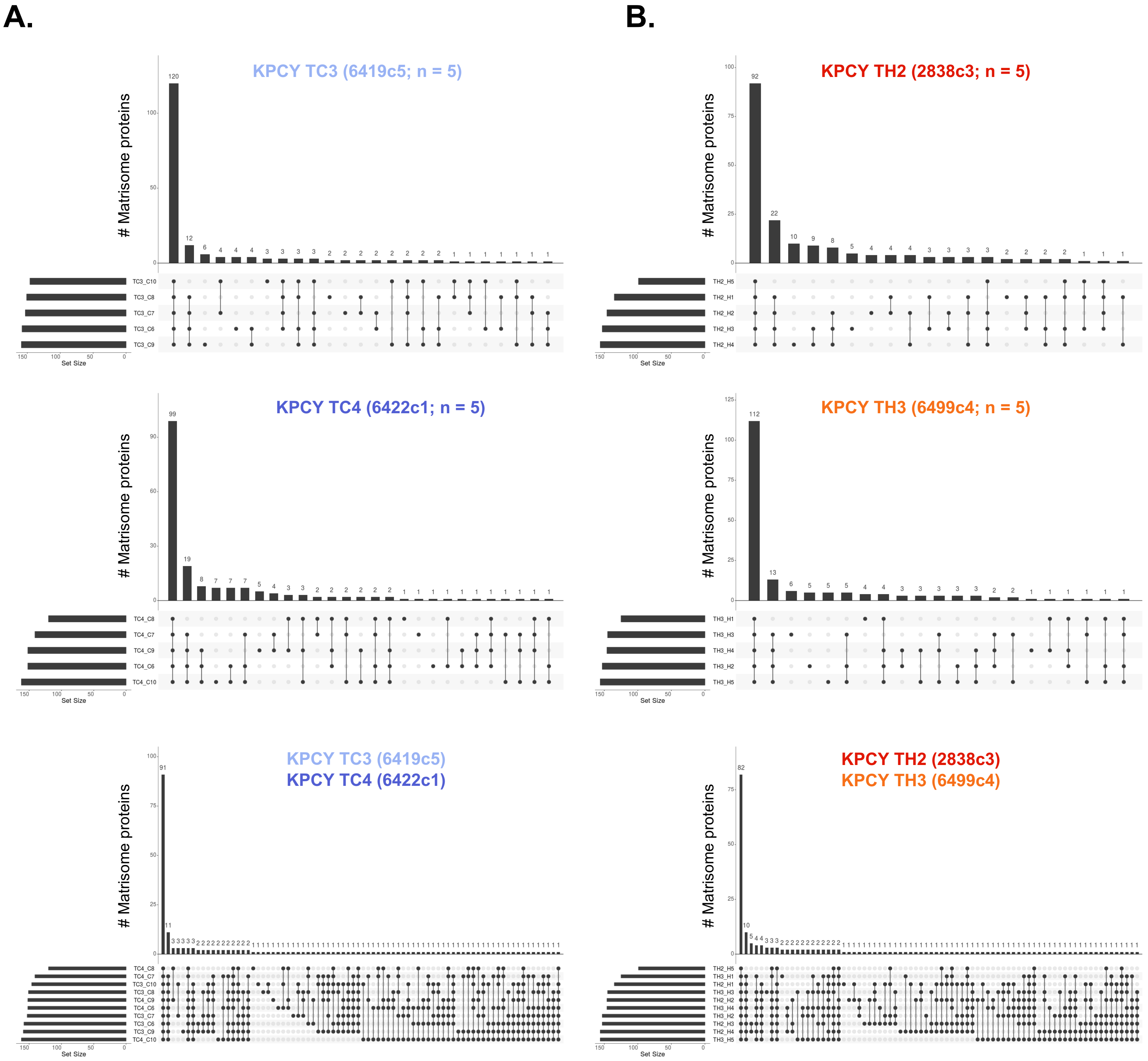

Supplementary Figure 3. Intragroup reproducibility of the proteomic output; *Related to Figure 2B and 2D.*

A. Upset plots represent the overlap between the matrisome proteins identified by mass spectrometry in CD8^{lo} TC3 (6419c5 cell line; top panel), TC4 (6422c1 cell line; middle panel), or across both TC3 and TC4 KPCY PDAC samples (lower panel).

B. Upset plots represent the overlap between the matrisome proteins identified by mass spectrometry in CD8^{hi} TH2 (2838c3 cell line; top panel), TH3 (6499c4 cell line; middle panel), or across both TH2 and TH3 KPCY PDAC samples (lower panel).
