# Supplementary material for: Identification of Pancreatic Ductal Adenocarcinoma Extracellular Matrix Signatures from In-Depth Proteomic Profiling that Correlate with Lymphocyte Infiltration
Source: Cancer Res Commun. 2026 Jun 5;6(6):1319–35. doi: 10.1158/2767-9764.CRC-25-0460 (PMC13236633; doi:10.1158/2767-9764.CRC-25-0460)
Supplement: Supplementary Figure 4 — Qualitative assessment of the distribution of proteins detected in differential abundance between CD8hi and CD8lo KPCY tumors using IHC [file crc-25-0460_supplementary_figure_4_suppsf4.pdf]

Supplementary Figure 4. Qualitative assessment of the distribution of proteins detected in differential abundance between CD8^{hi} and CD8^{lo} KPCY tumors using IHC

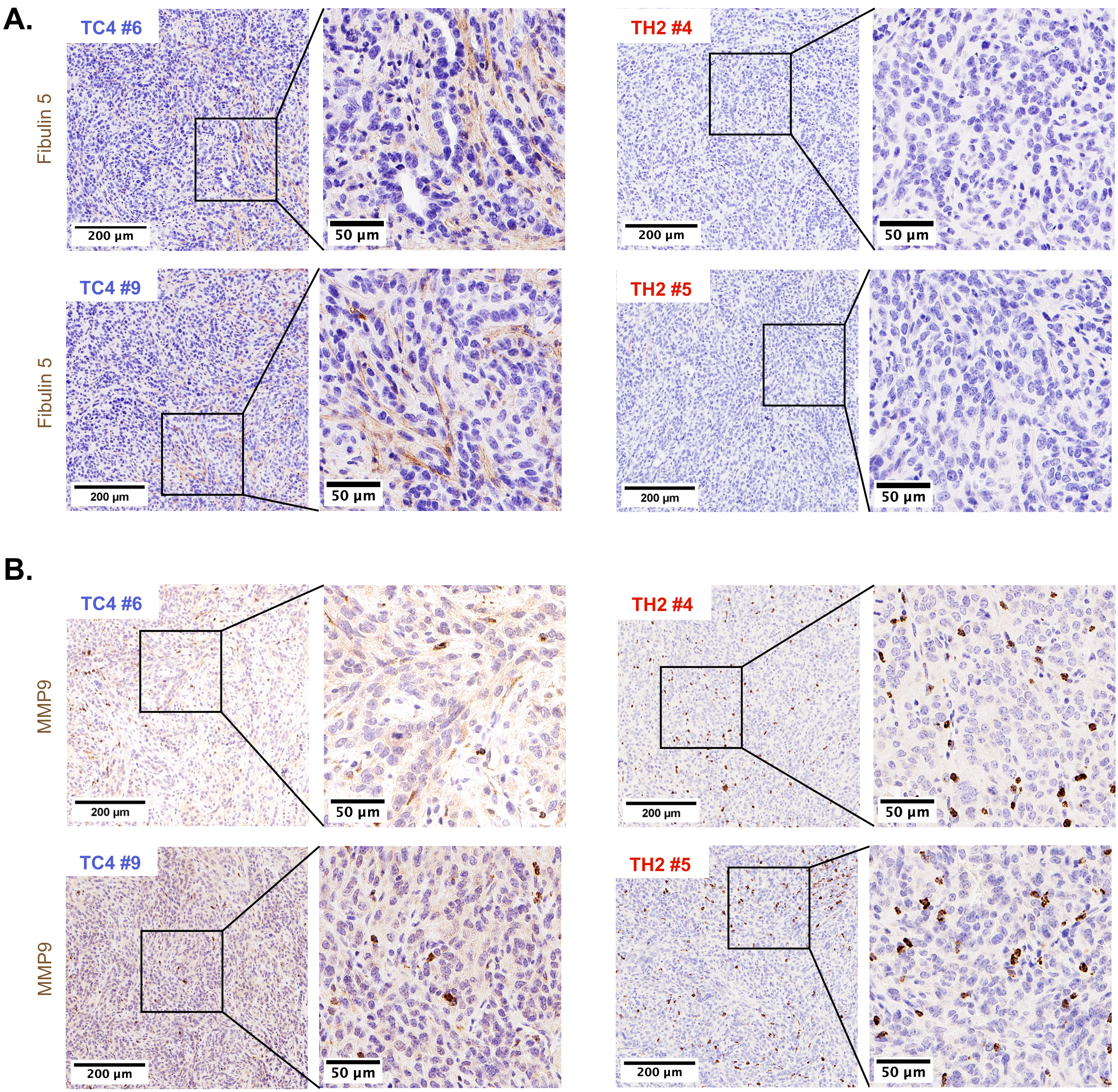

Supplementary Figure 4 (continued)

C.

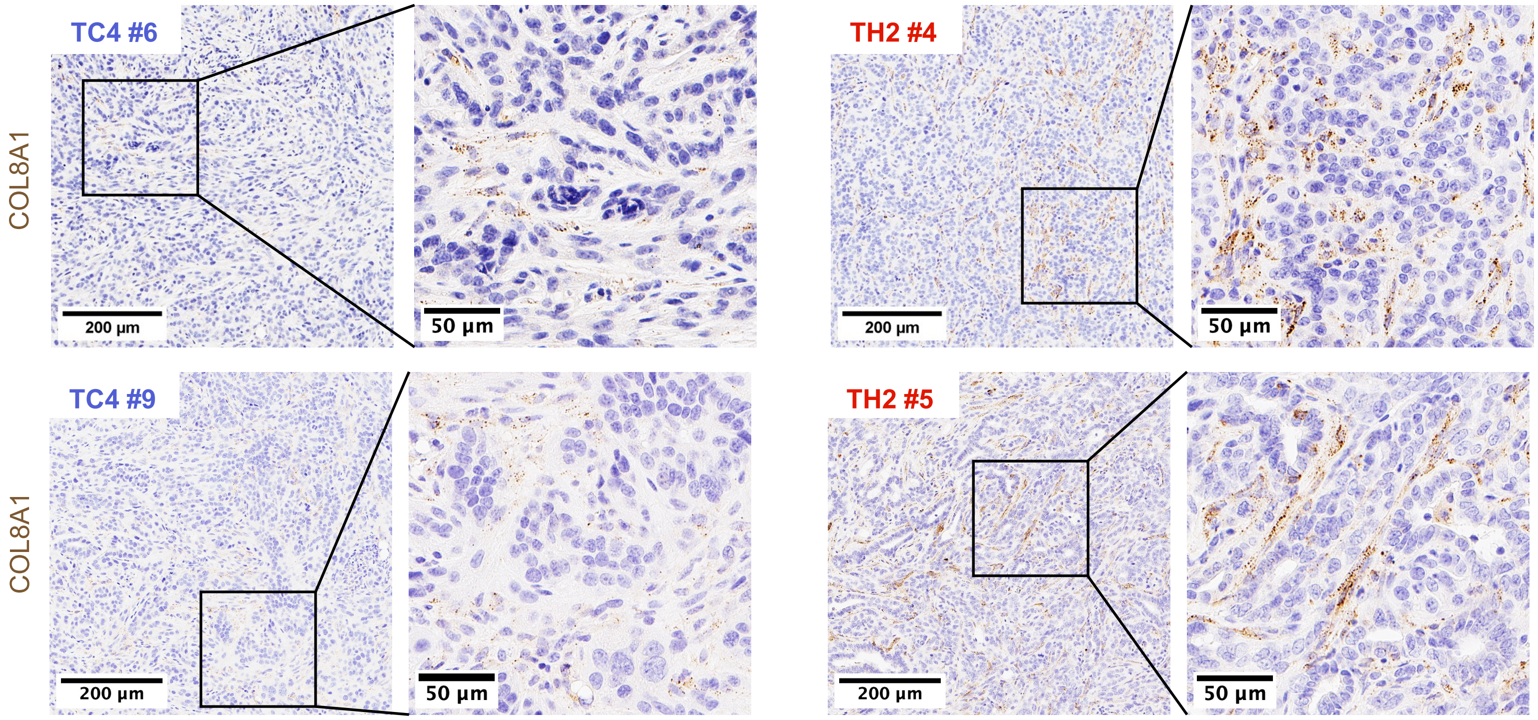

D.

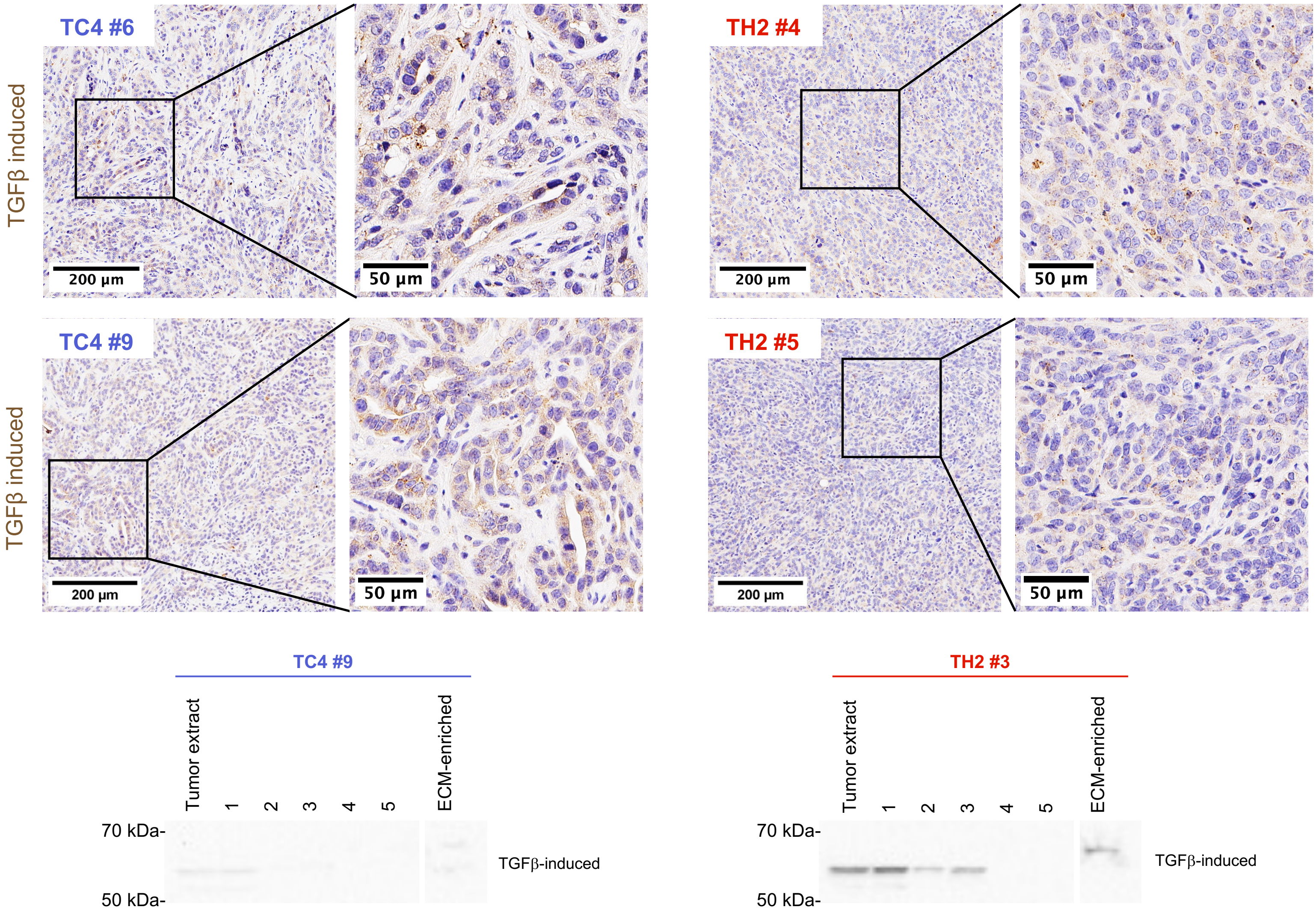

Supplementary Figure 4. Qualitative assessment of the distribution of proteins detected in differential abundance between CD8^{hi} and CD8^{lo} KPCY tumors using imunohistochemical staining
One section of each tumor sample, with 2 tumors per group (n=2), was stained using antibodies directed against proteins identified in the proteomic analysis:

A. Representative image of 5µm-thick sections from CD8^{lo} TC4 (*left*) and CD8^{hi} TH2 (*right*) tumors stained with an anti-fibulin 5 antibody, supporting the proteomic data and showing the presence of fibulin 5 in the ECM of CD8^{lo} tumors and its absence in the ECM of CD8^{hi} tumors (scale bar: 200µm). Higher-magnification insets are also provided (scale bar: 50µm).

B. Representative image of 5µm-thick sections from CD8^{lo} TC4 (*left*) and CD8^{hi} TH2 (*right*) tumors stained with an anti-MMP9 antibody confirming the proteomic data and showing the presence of MMP9 in the ECM of CD8^{lo} tumors (*left panels*) and its absence in the ECM of CD8^{hi} tumors (*right panels; note that the weak positive staining in TH2 tumor sections is intracellular*) (scale bar: 200µm). Higher-magnification insets are also provided (scale bar: 50µm).

C. Representative image of 5µm-thick sections from CD8^{lo} TC4 (*left*) and CD8^{hi} TH2 (*right*) tumors stained with an anti-collagen VIII (COL8A1) antibody, supporting the proteomic data and showing the absence of COL8A1 in the ECM of CD8^{lo} tumors (*left panels*) and its presence in CD8^{lo} tumors (scale bar: 200µm). Higher-magnification insets are also provided (scale bar: 50µm).

D. *Top and middle panels:* Representative image of 5µm-thick sections from CD8^{lo} TC4 and CD8^{hi} TH2 tumors stained with an anti-TGFβ induced antibody showing positive signals in both CD8^{lo} and CD8^{hi} tumors (scale bar: 200µm). Higher-magnification insets are also provided (scale bar: 50µm). *Bottom panels*: Immunoblots illustrate the abundance of TGFβ induced in protein fractions of increasing insolubility and show an enrichment of TGFβ induced in the ECM-enriched protein fractions of CD8^{hi} TH2 tumors (*right panel*) but not of CD8^{lo} TC4 tumors (*left panel*).
