# Supplementary material for: Identification of Pancreatic Ductal Adenocarcinoma Extracellular Matrix Signatures from In-Depth Proteomic Profiling that Correlate with Lymphocyte Infiltration
Source: Cancer Res Commun. 2026 Jun 5;6(6):1319–35. doi: 10.1158/2767-9764.CRC-25-0460 (PMC13236633; doi:10.1158/2767-9764.CRC-25-0460)
Supplement: Supplementary Table 1 — Antibody list [file crc-25-0460_supplementary_table_1_suppst1.pdf]

Supplementary Table 1. List of antibodies used in this study

Antibody target	Source	Catalog #	Research Resource Identifiers (RRID)	Host	Application	Concentration or Dilution
**Actin**	Hynes lab (MIT)	Clone 14-4	n/a	Rabbit	Western blot	1:5,000
**Collagen I (Col1a1)**	Sigma	AB765P	AB_92259	Rabbit	Western blot	0.5 µg/mL
**GAPDH**	Sigma	MAB374	AB_2107445	Mouse	Western blot	2.0 µg/mL
**Histone H4**	Sigma	05-858	AB_390138	Rabbit	Western blot	1:30,000
**Integrin β1**	Hynes lab (MIT)	ROH 210	n/a	Rabbit	Western blot	1:1,000
**αSMA**	Abcam	ab5694	AB_2223021	Rabbit	IHC; HIER	2.0 µg/mL
**Agrin**	Abcam	ab85174	AB_1860988	Rabbit	IHC; HIER Tris, pH 9	1.0 µg/mL
**CD31**	Cell Signaling	77699	AB_2722705	Rabbit	IHC; HIER Tris, pH 9	1:200
**CD4**	Cell Signaling	98986	Unavailable	Rabbit	IHC; HIER Tris, pH 6	1:400
**CD8**	Cell Signaling	98941	Unavailable	Rabbit	IHC; HIER Tris, pH 9	3.2 µg/mL
**Collagen VIII (Col8a1)**	Sigma	HPA053107	AB_2682046	Rabbit	IHC; HIER Tris, pH 6	1.0 µg/mL
**F4/80**	Cell Signaling	70076	AB_2799771	Rabbit	IHC; HIER Tris, pH 6	1:500
**Fibulin 5**	Abcam	ab109428	AB_10863582	Rabbit	IHC; HIER Tris, pH 9	0.7 µg/mL
**Ly6G**	Cell Signaling	87048	AB_2909808	Rabbit	IHC; HIER Tris, pH 9	1:200
**MMP9**	Abcam	ab38898	AB_776512	Rabbit	IHC; HIER Tris, pH 9	1.0 µg/mL
**TGFβ-induced**	Sigma	HPA017019	AB_2669511	Rabbit	IHC; HIER Tris, pH 6	0.2 µg/mL

** HIER: Heat-induced epitope retrieval*
